# Synthesis, Characterization, and Electrochemical Behavior of Ternary Ni–Co–Fe Prussian Blue Analogues

**DOI:** 10.1021/acsomega.5c05763

**Published:** 2025-08-19

**Authors:** Isabella Concina, Janna Attari, Farid Akhtar, Alessio Mezzi, Shujie You

**Affiliations:** † Department of Engineering Sciences and Mathematics, Luleå University of Technology, Luleå 97187, Sweden; ‡ 228703 CNR-ISMN, Strada provinciale 35d, n.9, Montelibretti, Roma 00010, Italy

## Abstract

We present a systematic investigation on the structural and physical features of a series of ternary Prussian blue analogues (t-PBAs) based on cobalt, nickel, and iron (Co/Ni ratio varied from 0 to 1), coupled with the study of their electrochemical performance as potential supercapacitors. The materials are prepared through the well-established coprecipitation method in water, proving it as a robust approach for a fine *on demand* modulation of their composition. The increase of the Co content rules the extent of crystallite sizes, as well as the strength of the bond in the iron-cyanide ligand, through internal charge exchange between these two transition metal ions. The relative amount of Co and Ni, on the other hand, significantly affects their electrochemical behavior: the formal potentials, the current generated under voltage scanning, the resistance to long bias solicitations, and the characteristics of galvanostatic charge–discharge are all affected by this parameter. A mixed charge storage control mechanism is observed for both capacitive and diffusive processes for the entire batch. The supercapacitive behavior is investigated as well, showing no obvious dependence on material composition, although fairly good performance in terms of capacitance and capacitance retention is recorded.

## Graphical abstract

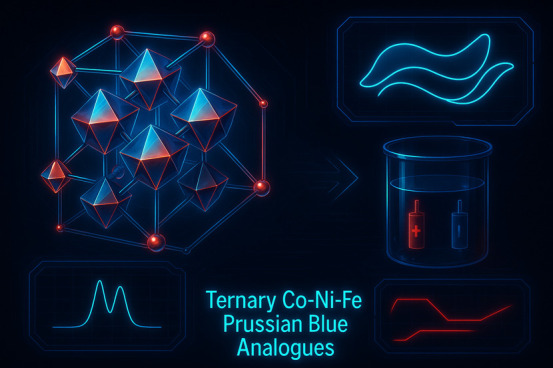

## 1 Introduction

Prussian blue analogues (PBAs) are currently regarded as an extremely promising class of materials to exploit in diverse applications, spanning from energy conversion and storage to water remediation through adsorption and photocatalysis, for which interesting results have been reported.^1–8^

This attention is ascribed to their unique structural features, self-healing capacity, open framework to insert/desert alkali ions, easiness of preparation, and electrochemical skills.^9^ Furthermore, when dealing with energy storage devices, they are being proved capable of operating in nonaggressive electrolytes,^6^ which definitely adds an asset to their exploitation in real scenarios, avoiding the corrosion damages at the device closures typical in batteries.

Although this class of materials has been known for almost three centuries, the research interest in their exploitation is relatively recent, and most literature discussion is based on binary analogues, where a different transition metal than iron is substituted in the PB structure (general formula: A_*n*_M_*y*_[M′(CN)_6_]*x*·H_2_O, where A is an alkali metal and M and M′ are transition metals).

Ternary PBAs (t-PBAs), in which a third transition metal ion is added to the structure, are rarely investigated. The literature lacks systematic investigations on the interplay of composition/structure/functional performance, while rationalizing this relation is a mandatory action if we want to retrieve precise information on how to efficiently design them.

In the present study, we prepared a series of five PBAs based on Ni and Co hexacyanoferrates through the classic coprecipitation approach, without the use of any surfactant, varying the Co-to-Ni ratio from 0 to 1.

The effect of different compositions on their structural, textural, and physicochemical features is discussed, followed by the analysis of their electrochemical behavior in quasi-neutral electrolyte (K_2_SO_4_) performed by cyclic voltammetry (CV) and galvanostatic charge–discharge (GCD) measurements, to evaluate their potential as supercapacitors.

We herein propose a classic inorganic chemistry study, where the analysis of material features is performed from a perspective in between coordination chemistry and solid state physics.

## 2 Experimental Section

### 2.1 Materials

All chemicals were purchased from Sigma-Aldrich and used without any further purification.

### 2.2 Synthesis of Ternary Prussian Blue Analogues

t-PBAs were prepared by a coprecipitation method at room temperature (RT). Stock solutions of K_3_[Fe­(CN)_6_] (0.04 M), Co­(NO_3_)_2_·6H_2_O (0.08 M), and Ni­(NO_3_)_2_·6H_2_O (0.08 M) in distilled water were prepared. A 50 mL of the K_3_[Fe­(CN)_6_] stock solution was poured in a glass and kept under magnetic stirring (450 rpm). Co­(NO_3_)_2_·6H_2_O and Ni­(NO_3_)_2_·6H_2_O solutions were then dropped in an alternate fashion with a dropping speed of 1 mL/min, in amounts such as to reach the following Co/Ni ratio: 0/1, 1/3, 1/1, 3/1, and 1/0.

### 2.3 Structural and Morphological Characterization

#### 2.3.1 X-ray Diffraction Analysis

Powder XRD patterns of the samples were recorded on a PANanalytical Empyrean instrument equipped with a PIXcel3D detector and operated at 40 kV and 45 mA using Cu Kα radiation. The obtained results were analyzed using High Score Plus software (vers.3.0.1). To calculate the cell parameter a, first the interplanar spacing *d* was calculated from Bragg’s law (*n*λ = 2*d* sin θ), considering *n* = 1 and λ = 1.54178 Å. Then, from Miller indices *h*, *k*, *l*, one obtains: $a=d\sqrt{h^{2}+k^{2}+l^{2}}$ . The error in the angle is given by the experimental spectral resolution (0.026°) and propagated to obtain the error in cell parameter *a*. Data were compared with the ICSD reference XRD database. Crystallite sizes were calculated using the Scherrer equation:^10^ $D=\frac{k\lambda }{\beta \cos\theta }$ , where *D* is the crystallite size, λ is the wavelength of the X-ray source (1.54178 Å), *k* is a shape factor (0.94 in the present case), β is the full width at half-maximum, and θ is the Bragg angle in radians.

#### 2.3.2 Scanning Electron Microscopy and Energy Dispersive X-ray Spectroscopy

Energy-dispersive X-ray spectroscopy (EDX) was carried out in a scanning electron microscope (JSM-IT300). AZtech Oxford software was used for the extraction of the EDX results from the raw data. The appropriate amount of the samples was mounted on a metal plate by Leit-C conductive carbon (Plano GmbH, Wetzlar, Germany) prior to insertion into the microscope.

### 2.4 X-ray Photoelectron Spectroscopy

The surface chemical composition of the samples was investigated by an ESCALAB MkII apparatus equipped with a standard twin-anode Al/Mg X-ray source and five-channeltron detection device. To avoid overlap between the NiLMM Auger signals and Co 2p_3/2_ core level signal, the Mg X-ray source (*h*ν = 1253.6 eV) was preferred to the Al X-ray source (*h*ν = 1486.6 eV). All samples were mounted by fixing the powder to a bidhesive carbon disc, taking care to spread the powder to completely cover the carbon disc. All spectra were registered operating at a constant pass energy of 40 eV; the accuracy of BE value was ±0.1 eV, while the BE scale was calibrated by positioning the contribution of the adventitious carbon at 285.0 eV. All spectra were collected and processed by Avantage v.5.9 software. More details are reported elsewhere.^11^

### 2.5 Textural Analysis

The Brunauer–Emmett–Teller (BET) surface area was measured through a surface area analyzer (Gemini VII 2390, Micromeritics, USA). For the measurements, a minimum of 100 mg of sample mass was degassed under a high vacuum overnight at 150 °C (sample Co/Ni 1/1) and at 200 °C (samples Co/Ni 1/3, 3/1, and binary analogues). The degassed specimens underwent nitrogen (N_2_) adsorption at isothermal conditions using a liquid nitrogen bath (−196 °C). The BET equation was used to calculate the surface area with *P*/*P*_0_ in the range of 0.05–0.30. Considering N_2_ adsorbed and the shape of the adsorption isotherms (type IV), the pore size and average pore volume were determined using the BJH method applied to the desorption part. This method is based on the modified Kelvin equation, assuming cylindrical, nonoverlapping pores and the condition that at *P*/*P*_0_ = 1, all the pores are filled with liquid. However, the validity of this method becomes questionable if any of these assumptions are not applicable. Notably, it has been demonstrated that for narrow mesopores smaller than 10 nm, the BJH method underestimates the pore size by up to 20–30%.^12,13^

### 2.6 Fourier Transform Infrared Spectroscopy

A diffuse reflectance Fourier transform infrared (DRIFT) spectroscopic technique was performed using a Bruker VERTEX 70v Fourier transform infrared (FTIR) spectrometer (Germany). Each sample was analyzed in the range 4000–400 cm^–1^ by averaging 128 scans at a spectral resolution of 2 cm^–1^.

### 2.7 Thermal Analyses

Thermal analyses were recorded in a TG-DTA, TG-209F3, NETZSCH, Germany analyzer from 20 to 500 °C, at 5 °C/min, under nitrogen flow.

### 2.8 Electrochemical Measurements

Each PBA (5 mg) was mixed with mesoporous carbon (2.5 mg) and dispersed in 5 mL of ethanol (containing 0.6% weight PTFE) to obtain a slurry. This latter was sonicated at RT for 90 min. A volume of 40 μL of the slurry was drop cast on a glassy carbon (GC) electrode (3 mm diameter) and left to dry in ambient conditions (weight of active material deposited: 3.883 × 10^–2^ mg).

Electrochemical measurements were carried out in a three-electrode configuration, featuring standard calomel as the reference electrode (RE), graphite as the counter electrode (CE), and the PBAs on glassy carbon as the working electrode (WE). K_2_SO_4_ or Na_2_SO_4_ in water (0.5 M) was used as the electrolyte. All the measurements were carried out in a multipurpose electrochemical station (Solartron Analytical, ModulabXM). CV was recorded by scanning the potential from 0 to +1 V vs SCE at different rates (100, 50, 25, and 5 mV/s) for 5 cycles. The retention of performance was evaluated by scanning the WE in the same potential range at 100 mV/s for 2000 cycles. Specific capacitance was evaluated from the CV curves according to the following equation: $C=\frac{Q}{\Delta V\times w}$ , where *Q* is the charge ( $Q=\frac{Area}{2\times \nu }$ , with ν being the scan rate), Δ*V* is the voltage window, and *w* is the weight of the active material.

GCD measurements were carried out by imposing a constant current (from 25 to 1000 mA/g) and monitoring the potential attained by the WE over time.

## 3 Results and Discussion

### 3.1 Structural and Textural Characterization

Results from the XRD analysis are reported in Figure 1 and Table 1.

**Figure 1:**
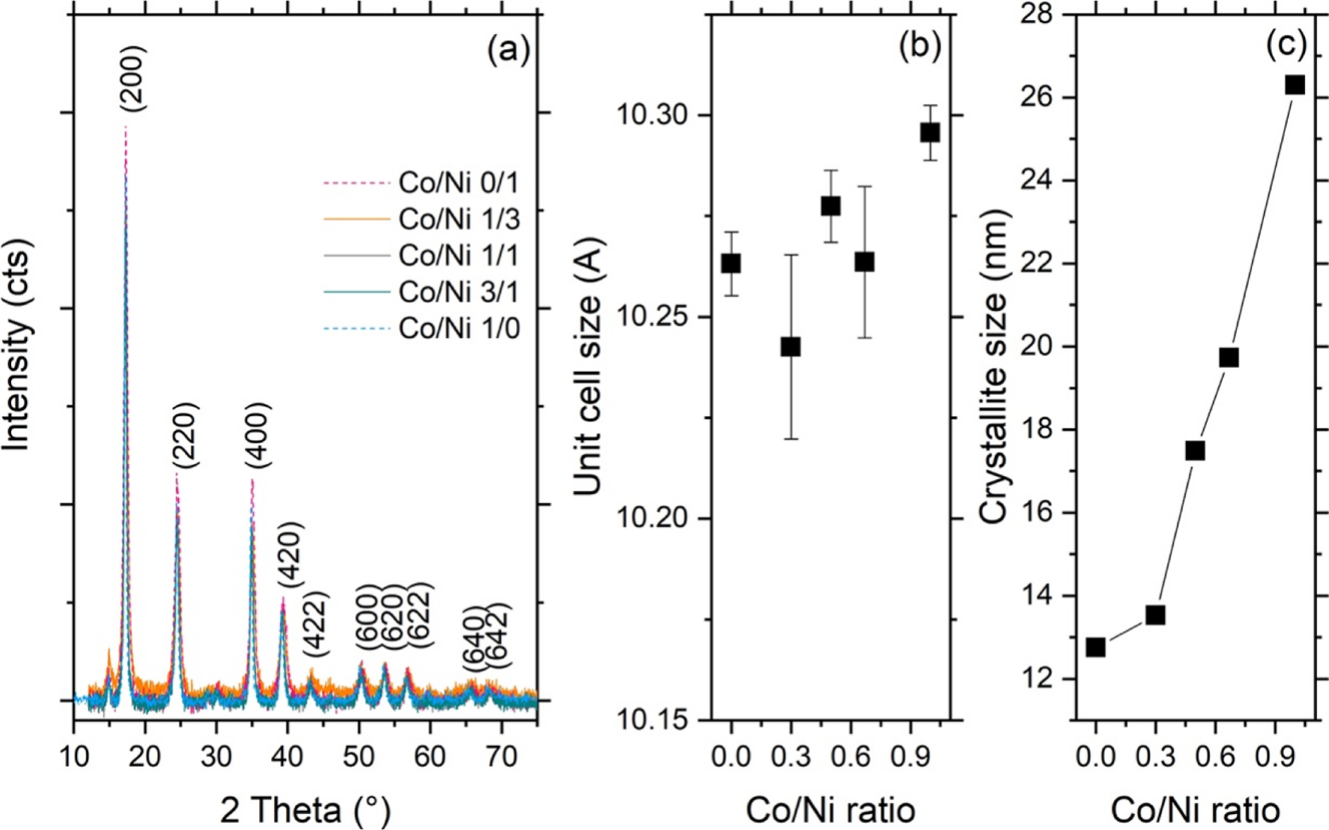
(a) XRD patterns of the prepared materials. Binary PBA containing only Ni or Co are plotted in dashed lines for clarity purposes. Miller indexes of the crystalline planes are reported. (b) Unit cell size and (c) crystallite size as a function of the nominal Co/Ni ratio.

**Table 1:** XRD Parameters as Calculated from XRD Patterns

Co/Ni ratio	sample label	unit cell size (Å)	crystallite size (nm)
0/1	NiHCF	10.26 ± 7.91 × 10^–3^	12.75
1/3	T2	10.24 ± 2.29 × 10^–2^	13.53
1/1	T1	10.28 ± 8.93 × 10^–3^	17.48
3/1	T4	10.26 ± 1.88 × 10^–2^	19.73
1/0	CoHCF	10.29 ± 6.85 × 10^–3^	26.30

Figure 1a shows the XRD patterns of the prepared materials: all peaks of PBAs are indexed with a typical face-centered cubic structure with the space group *Fm*3̅*m*.^14^ The unit cell parameter *a* was found not to change significantly with the composition (Figure 1b), showing fluctuations between the smallest (binary Ni analogue) and highest (binary Co analogue) value of the batch, which were found in line with previously reported sizes.^15^ On the contrary, increasing the Co/Ni ratio significantly affected the extension of coherent domains in the materials (Figure 1c): crystallite sizes monotonically increased with the amount of Co included in the PBAs, reaching their maximum in the case of the binary Co analogue (Co/Ni ratio 1/0).

This finding indicates different kinetics in the nucleation/growth process, depending on the relative amount Co/Ni: the presence of Ni induces a faster nucleation, at the expense of crystal growth.

Another important parameter used to determine the structure of PBAs is the amount of water molecules included in the lattice. These inorganic polymers indeed contain different kinds of water molecules, usually classified as (a) “zeolitic” water (sitting in the cavities formed by the M–CN–M framework), whose ideal number was quantified as eight molecules per unit cell;^16^ (b) coordinated water (replacing the vacant [Fe­(CN_6_)] octahedra), whose ideal amount is also reported to eight molecules per unit cell;^16^ and (c) hydrogen bound water. Thermal analyses are an ideal tool in this respect, allowing for a differentiation between interstitial and coordinated water molecules, which are released from the materials at different temperatures. Thermal analyses of the PBAs under investigation and the amount of zeolitic and coordinated water molecules are shown in Figure S1 and Table S1 in the Supporting Information. The temperature range of interest for dehydration is between RT and 180 °C, followed by sample degradation at higher temperatures. A rather similar behavior upon heating was observed for all the samples: a significant weight loss (about 20%) was recorded up to about 200 °C, accompanied by an endothermic process (visible in the DSC patterns reported in Figure S1b), ascribable to the release of water. Above this temperature, material decomposition starts: attempts to identify the effluents from the thermobalance were previously done in literature for binary analogues,^17,18^ and in the present study we did not investigate the decomposition products.

The analysis of thermal gravimetric curves allowed us to quantify the amount of water molecules in the samples (Table S1 in the Supporting Information). We did not find a monotonic trend of the water content as a function of the Co/Ni ratio, but this was not surprising, considering that it is well-known that the amount of water can significantly vary in PBAs, often leading in literature to an unclear quantification.^19^ However, we observed that the Ni and Co analogues featured the highest and lowest amounts of coordinated water molecules, respectively: the hexaaquanickel­(II) ion, [Ni­(H_2_O)_6_]^2+^, resulted less prone to renounce water as a ligand than its Co homologue, [Co­(H_2_O)_6_]^2+^, which also explains the longer degree of order in the final material.

EDX analysis (reported in the Supporting Information, Figure S2) was used to determine the composition of the materials in terms of metal content, which, combined with the information obtained from thermal analyses, led to the determination of the experimental stoichiometry of the materials (Table 2).

**Table 2:** PBA Composition and Empirical Formulas, as Retrieved by Combining the Information Obtained from EDX and Thermal Analyses

Co/Ni ratio	sample label	K (at %)	Fe (at %)	Co (at %)	Ni (at %)	formula	experimental Co/Ni ratio
0/1	NiHCF	2.12	11.4	0	17.4	K_0.37_Ni_3.04_[Fe(CN)_6_]_2_ × 8.9 H_2_O	0
1/3	T2	0.76	4.96	2.31	5.30	K_0.30_Co_0.93_Ni_2.14_[Fe(CN)_6_]_2_ × 7.9 H_2_O	0.44
1/1	T1	0.57	3.18	2.26	2.20	K_0.36_Co_1.42_Ni_1.38_[Fe(CN)_6_]_2_ × 6.4 H_2_O	1.03
3/1	T4	1.11	5.41	5.48	2.21	K_0.40_Co_2.02_Ni_0.82_[Fe(CN)_6_]_2_ × 7.1 H_2_O	2.46
1/0	CoHCF	2.22	5.12	7.89	0	K_0.80_Co_3.00_ [Fe(CN)_6_]_2_ × 5.5 H_2_O	∞

The relative amount of Fe^II^ and Fe^III^ was also investigated by means of FTIR spectroscopy. By analyzing the stretching vibration of the C≡N ligand, it is indeed possible to calculate the ratio between the two oxidation states. This vibration is present as a doublet, and the position of the two peaks depends on the oxidation state of the iron ion: the peak at higher energy pertains to the bond with Fe^III^, and the peak at lower energy to the bond with Fe^II^.

Figure 2 shows the FTIR spectra of the prepared materials (a complete correlation table is provided in the Supporting Information, Table S2). Significant displacement (about 60 cm^–1^) from the C≡N stretching position of iron precursor K_3_[Fe­(CN)_6_] (2119.6 cm^–1^) was recorded in all cases: the observed energy is indeed coherent with that recorded for PB (2162 and 2062 cm^–1^), used here as the benchmark specimen for the series (FTIR spectra and correlation tables for both the precursor and PB can be found in the Supporting Information, Figure S3 and Table S4, respectively).

**Figure 2:**
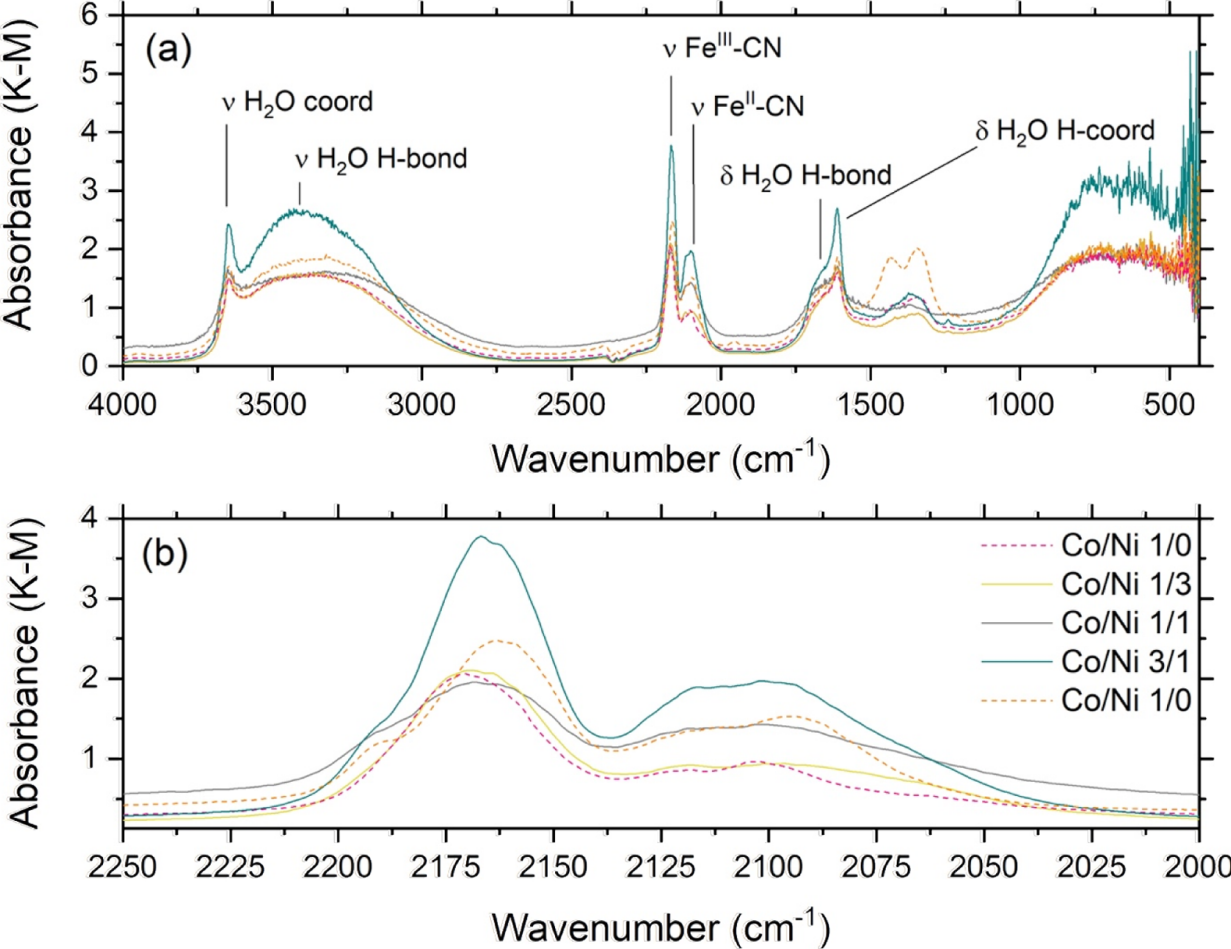
(a) FTIR spectra of the prepared materials. (b) Detail of the region pertaining to the C≡N stretching.

In the spectra pertaining to both the binary and ternary analogues, the appearance of the vibration modes related to the stretching of the C≡N ligand attached to Fe^III^ and Fe^II^ was observed as a doublet, between 2095 and 2219 cm^–1^, depending on the specimen identity. This indicates that the reaction with a second transition metal induces charge transfer to the iron center. The phenomenon is well-known in PB,^20^ for which, however, the charge cross-talk between the two iron centers, bound to the carbon and nitrogen atoms of the cyanide ligand, is faster than the resolution of vibrational spectroscopy at RT: C≡N stretching vibration thus appears as an asymmetric broad band with a tail toward lower energies (see Figure S3 in the Supporting Information). On the contrary, when a second (and then a third) transition metal ion substitutes the iron atom bound to the carbon in the cyanide ligand, inducing a charge transfer on the Fe^III^ atom, the C≡N stretching vibration becomes a doublet, whose energies change as a function of the Co/Ni ratio (Figure 3a,b and Table 3). Specifically, we observed a decreasing trend in the strength of the bond N≡C–Fe^III^ with the increase of the Co content, accompanied by a concomitant increase of the strength of the bond with Fe^II^, which significantly increased when Co was added to the structure, without any relevant difference among the t-PBAs including this transition metal ion. These findings are ascribable to the tendency of Co^II^ to delocalize one electron toward Fe^III^ through the bridging cyanide, which becomes more important with the increase of Co amount, enhancing the back-bonding character of the ligands.

**Figure 3:**
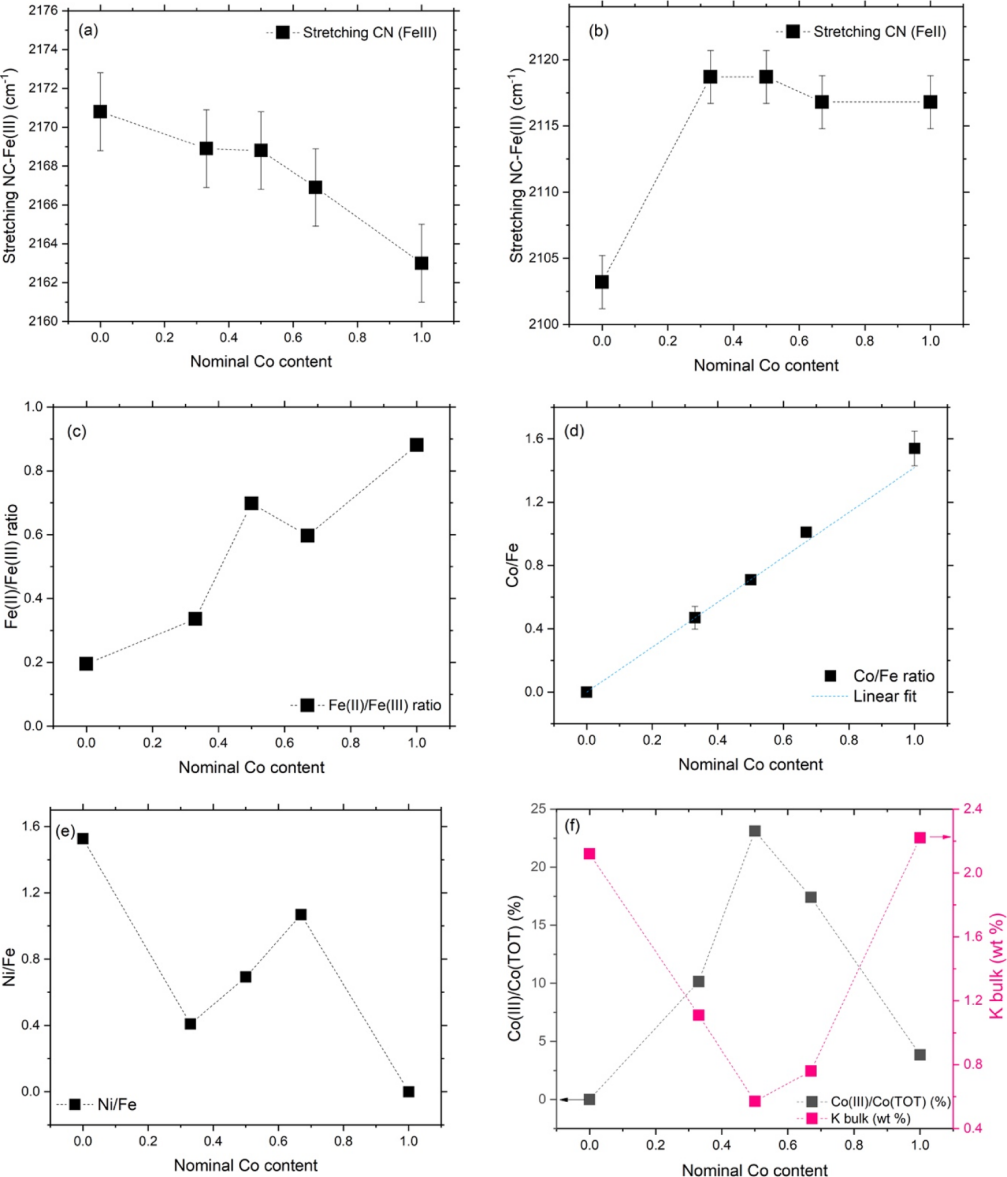
Influence of the nominal Co content on some properties of the PBAs under investigation. Changes in the energy of vibration of the stretching of CN ligand with (a) Fe­(III) and (b) Fe­(II). (c) Fe­(II)/Fe­(III) ratio as retrieved by FTIR spectroscopy. Relative amounts of Co (d) and Ni (e) to iron, as retrieved from EDX analysis. (f) Amount of Co­(III) on the total Co content (black markers) and amount of potassium (pink markers).

**Table 3:** Stretching Vibrations of Cyanide Group and Fe­(II)/Fe­(III) for the Prepared Samples

Co/Ni ratio	sample label	ν_C≡N_ (Fe (III)) (cm^–1^)	ν_**C**≡**N**_ (Fe (II)) (cm^–1^)	Fe(II)/Fe(III) ratio
0/1	NiHCF	2170.8	2103.2	0.195
			2096.5	
1/3	T2	2168.8	2118.7	0.336
			2096.5	
1/1	T1	2168.8	2118.7	0.698
			2102.3	
3/1	T4	2166.9	2116.8	0.597
			2101.3	
1/0	CoHCF	2163	2116.8	0.881
			2095.5	

This is also coherent with the nature of cyanide as a π-acceptor ligand in antibonding orbitals, supporting this electron transfer, thus taking the material to the maximum field stabilization in low spin complexes (both ions tend to a d^6^ configuration).

The ratio of Fe^II^/Fe^III^ for the different analogues was also calculated from the IR spectra by peak deconvolution and integration (results reported in Figure 3c): the amount of Fe^II^ was observed to increase with the increase in the Co content, indicating once again the transfer of one electron from Co­(II) to Fe­(III), resulting in the presence of Co^III^ and Fe^II^ in all the Co-containing analogues.

It was relevant to notice that the Co-to-iron ratio (considering all the oxidation states of the two metals) followed a perfect linear trend as a function of the nominal Co content (Figure 3d), indicating that (i) the synthetic approach was successful and suitable for the task and (ii) the increase in the amount of Fe^II^ is a direct consequence of the addition of Co. Furthermore, the evaluation of the ratio between Co in oxidation state + III to the total amount of Co showed a hill-shaped trend, nicely matching the valley-shaped trend of the amount of potassium (Figure 3f), confirming the role of the charge neutrality guardian of this latter in the ternary analogues. The higher the amount of Co­(III), indeed, the lower the potassium content revealed by EDX analysis, and viceversa. On the other hand, in the case of the binary Ni analogue, we found a quite high amount of potassium in the bulk and the lowest Fe^II^/Fe^III^ ratio.

We finally quantify the surface area, pore volume, and pore size (results reported in Table 4), which might be critical parameters in functional performance. The isotherms highlighted the presence of micro-, meso-, and macro-pores. No obvious trend with material composition was identified. This apparently anomalous behavior is not unusual in literature, where quite different values are reported for NiHCF (around 270 m^2^/g) and CoHCF, for which rather a broad spread in values can be found, depending on the specific characteristics of the specimens, ranging from 134 m^2^/g^21^ up to 572 m^2^/g.^22^ Concerning t-PBAs, very little information is found in literature about this parameter.^23^ In the present investigation, we determined a surface area for the t-PBAs of 266.19 m^2^/g for the analogue with equal amounts of Ni and Co, which, however, attained the highest values for pore volume and size, and much higher values (>400 m^2^/g) for the analogues with different Co/Ni ratios.

**Table 4:** Results from BET Analysis

Co/Ni ratio	sample label	BET surface area (m^2^/g)	pore volume (cm^3^/g)	pore size (Å)
0/1	NiHCF	279.26	0.31	44.0
1/3	T2	400.92	0.29	42.5
1/1	T1	266.19	0.37	67.9
3/1	T4	466.69	0.33	59.1
1/0	CoHCF	339.43	0.06	29.51

### 3.2 Surface Analysis

Surface elemental composition of the samples was investigated by X-ray photoelectron spectroscopy (XPS). The presence of hexacyanoferrate was confirmed by the N 1s and Fe 2p_3/2_ peaks, positioned at BE = 398.3 and 710.0 eV, respectively (Table 5).^11^ The ternary analogues were characterized by similar chemical composition, except for the Co and Ni, as expected, whose atomic ratio Co/Ni changed following approximately the expected value from the preparation, with a slight excess of Co (Co/Ni as high as 1.35) for the sample with nominal ratio 1/1, 0.6 (for that with nominal ratio 1/3), and 3.7 (for the nominal ratio 3/1). Figure 4 shows the comparison of Fe 2p, Co 2p, and Ni 2p_3/2_ signals. The 2p core level of these elements is characterized by the typical doublet 2p_3/2_–2p_1/2_, due to the well-known spin–orbit coupling, which takes place during the photoemission process.

**Table 5:** BE Value and XPS Quantitative Analysis

			C 1s			Fe 2p_3/2_		K 2p_3_	N 1s			O 1s	Co 2p_3/2_	Ni 2p_3/2_
Co/Ni ratio	sample label		C–C	–CN	–COOH(R)	Fe(2+)	Fe(3+)	K(1+)	–CN	–NH2	–NO	H2O	Co(3+)	Ni(2+)
0/1	NiHCF	BE (eV)	285.0		287.4		710.2		398.5		401.0	533.0		856.8
		At. (%)	51.6		3.9		7.2		23.9		1.5	6.5		5.5
1/3	T2	BE (eV)	285.0	287.2	288.7		710.1	293.9	398.3	400		532.8	782.7	856.5
		At. (%)	54.1	7.0	1.0		3.9	0.5	21.6	1.3		7.0	1.3	2.2
1/1	T1	BE (eV)	285.0	286.8	288.2		710.0	293.8	398.3	400.2		532.6	782.4	856.4
		At. (%)	51.2	7.9	2.0		4.0	0.8	23.2	0.8		6.1	2.3	1.7
3/1	T4	BE (eV)	285.0	286.8	288.3	708.5	710.3	293.8	398.4	400.1		532.7	782.4	856.6
		At. (%)	52.3	7.5	1.9	1.1	2.7	0.8	22	1.7		5.9	3.3	0.9
1/0	CoHCF	BE (eV)	285.0	287.4		708.7	710.2	293.8	398.5		401.1	532.4	782.6	
		At. (%)	46.3	5.3		2.6	5.1	0.7	25.8		2.4	4.8	7.0	

**Figure 4:**
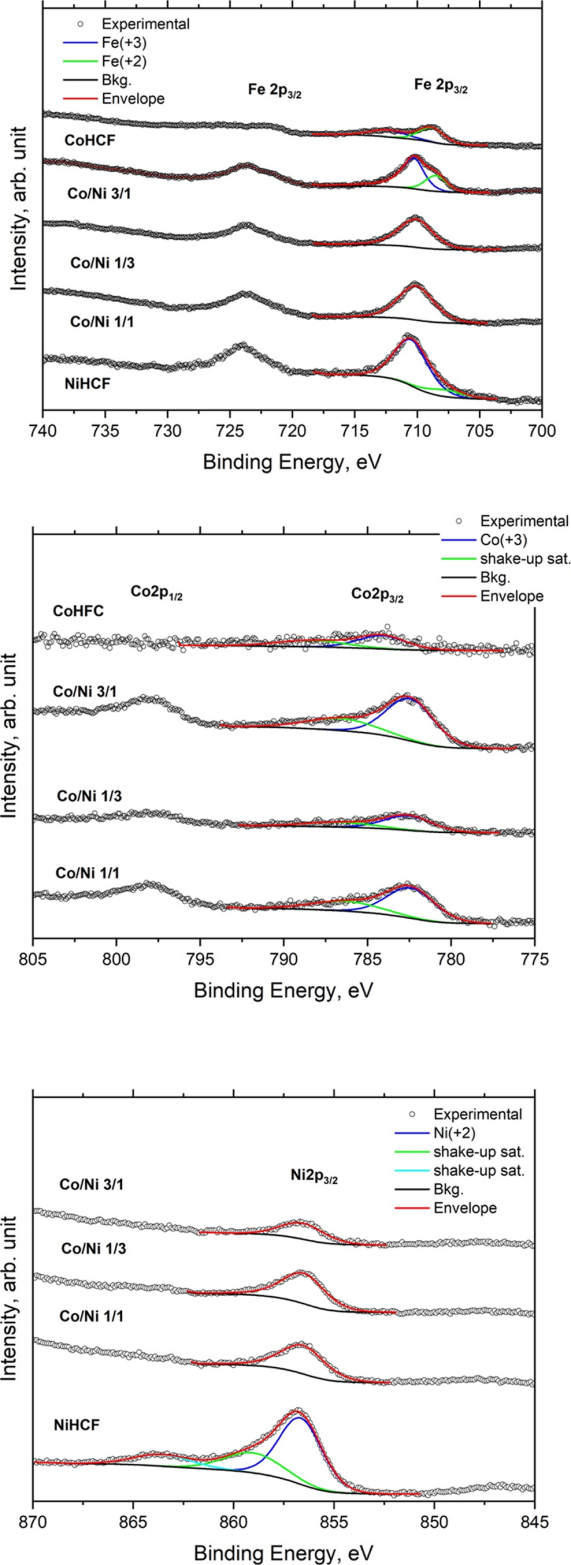
Comparison of the Fe 2p, Co 2p, and Ni 2p_3/2_ XPS spectra. CoHCF: binary Co analogue; NiHCF: binary Ni analogue.

Generally, p_3/2_ is considered for the assignment of the chemical state of the investigated element. In Table 5, it is possible to note all samples are characterized by Co and Ni in their +3 and +2 oxidation states in the cyano complex,^11^ respectively. As it concerns Fe 2p_3/2_, the peak-fitting analysis for the sample with Co/Ni 3/1 showed the presence of Fe in the +2 oxidation state.

Figure 5 shows a comparison of material composition in terms of metal content (reported as atomic percentage) in the bulk (Figure 5a) and on the surface (Figure 5b). Coherent trends between bulk and surface composition were observed for all the metal ions, with an increase (decrease) in the Co (Ni) content as a function of the amount of Co progressively inserted in the structure and an associated smooth increase in the amount of K. The amount of iron in the bulk (Figure 5a) showed a small pit in the monotonic increase (as a function of Co content), observed for the sample with a Co/Ni ratio 1/1, while an almost constant amount was recorded on the surface (Figure 5b).

**Figure 5:**
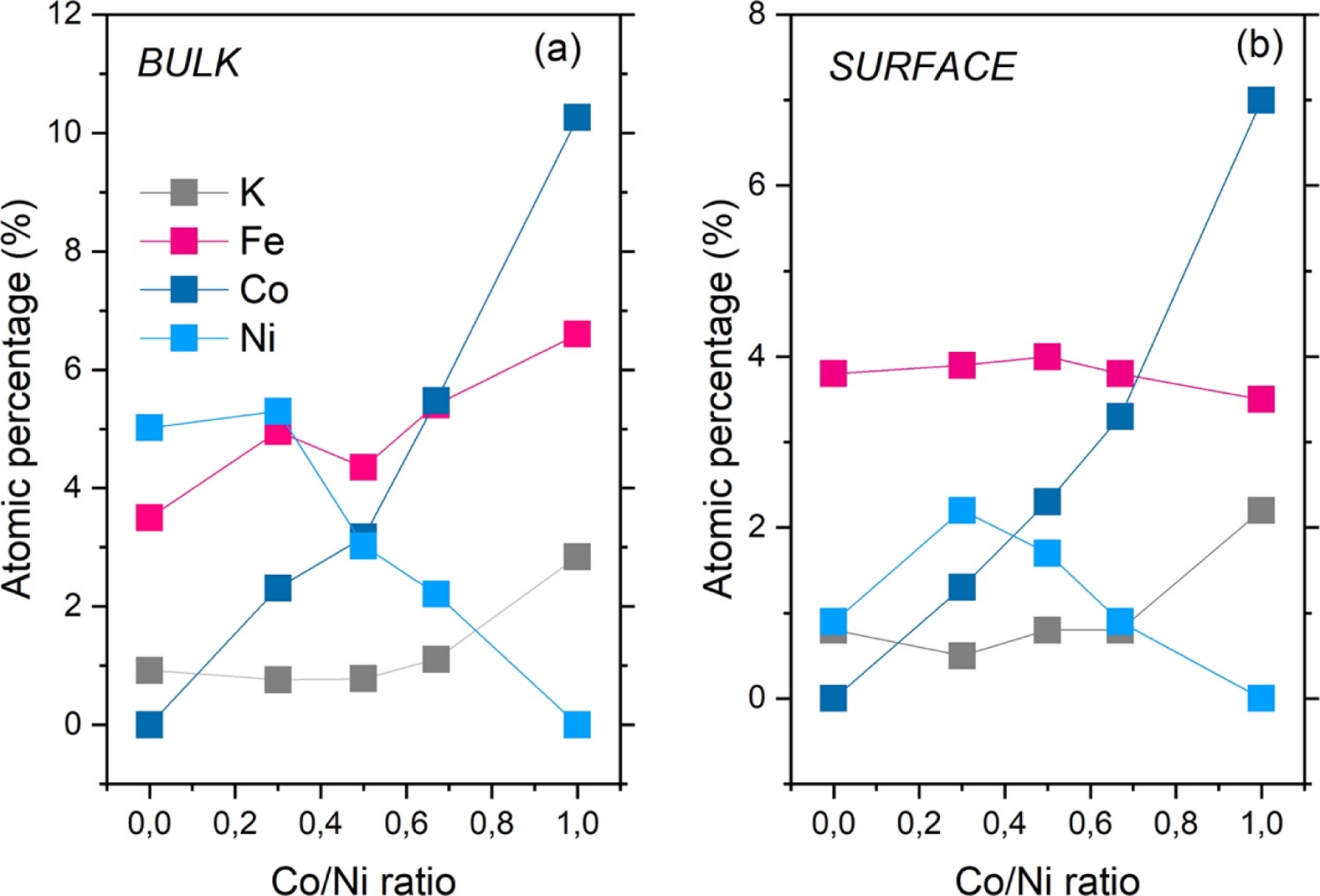
Bulk (a) and surface (b) atomic percentage of metals in the prepared PBAs as a function of the nominal Co/Ni ratio. Markers are experimental points; lines are guide for the eye.

### 3.3 Diffuse Reflectance Analysis

Figure 6 reports the diffuse reflectance spectra and the retrieved relevant energy parameters. All the PBAs showed similar spectral features, which can be analyzed in the light of analogy with coordination chemistry. Specifically, the valley centered around 33,000 cm^–1^ is ascribed to the octahedral splitting energy of the d-orbitals. This energy was found to depend on material composition (Figure 6b), with Co being the dominating factor in determining it. Specifically, the higher the Co amount, the higher the energy splitting.

**Figure 6:**
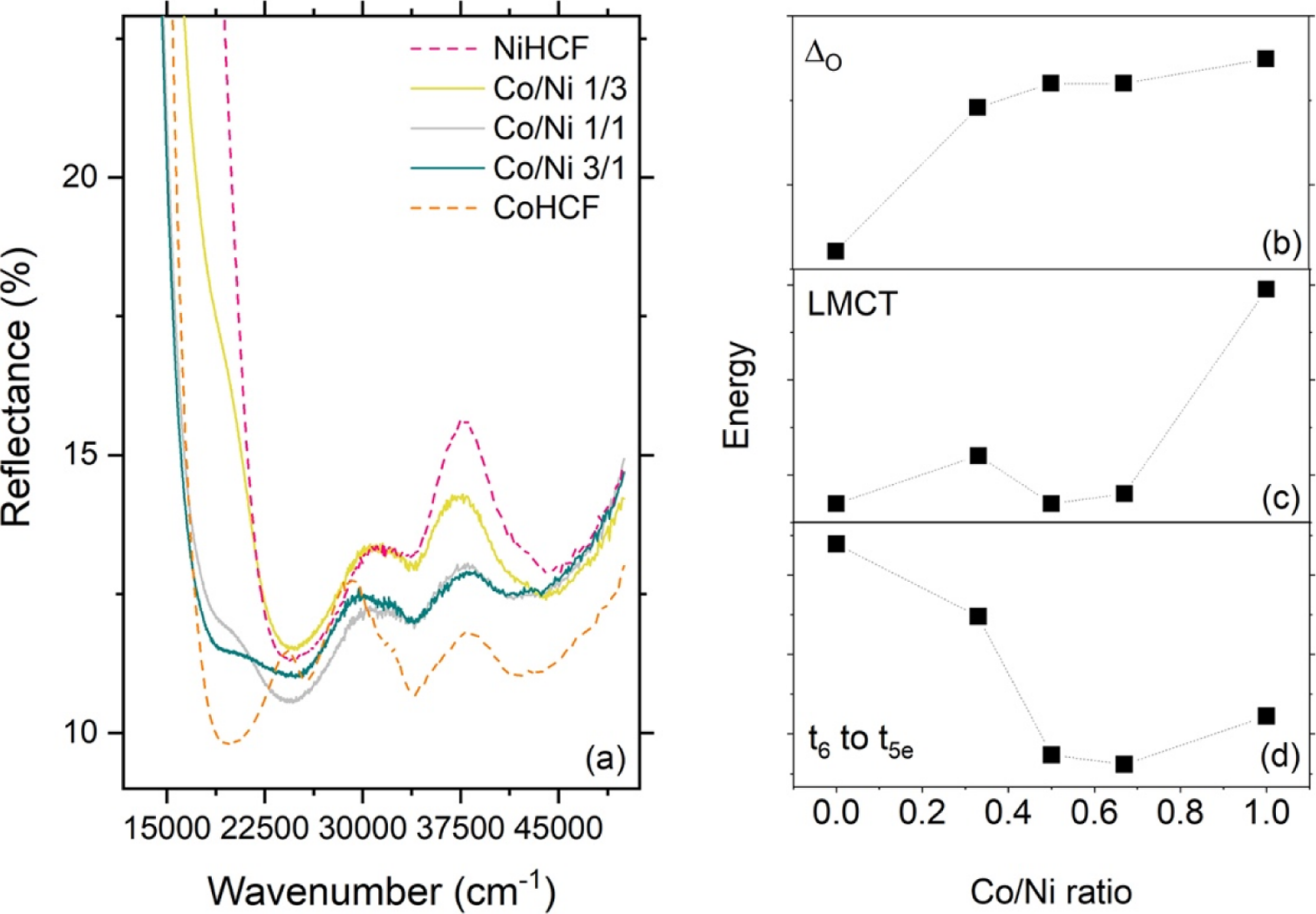
(a) DRS spectra of the prepared samples; energy of (b) octahedral delta, (c) ligand-to-metal charge transfer, and (d) t_6_ to t_5e_ transition as retrieved from DRS measurements.

The other two signals appear in all the spectra: a first valley around 24,000 cm^–1^ (ascribed to ligand-to-metal charge transfer), and another centered around 43,000 cm^–1^, associated to the lowest crystal field transition energy t_6_ → t_5e_ of the [Fe­(CN)_6_]^4–^ complex.^24,25^

Specimens with Co displayed another spectral characteristics, at about 19,000 cm^–1^, visible as a valley in the binary Co analogue and as a shoulder in the ternary samples: this signal is due to the transition from the ground state to an excited state in which an electron is shuttled from Fe^II^ to Co^III^ and is indeed absent in the binary Ni analogue, which is not available to further oxidation.^24^

### 3.4 Electrochemical Measurements

#### 3.4.1 Cyclic Voltammetry

Cyclic voltammetric analysis in K_2_SO_4_ (scan rate 100 mV/s) is shown for all of the analogues in Figure 7a (CV curves at different scan rates for each sample are reported in Figure S4 in the Supporting Information). The redox peaks are attributed to the redox reaction undergone by the hexacyanoferrate unit. Positive currents associate with the oxidation of the analogues and related release of the alkali metal from the matrix, while negative currents are due to the reduction Fe^III^ → Fe^II^ and concomitant uptake of the alkali metal. The redox processes were reversible for both the binary and ternary analogues, but it was noteworthy that different compositions are associated with different formal potentials *E* ^0^′ (Figure 7b), calculated as the midpoint potential of the anodic and cathodic peaks, as reported in Table 6.

**Figure 7:**
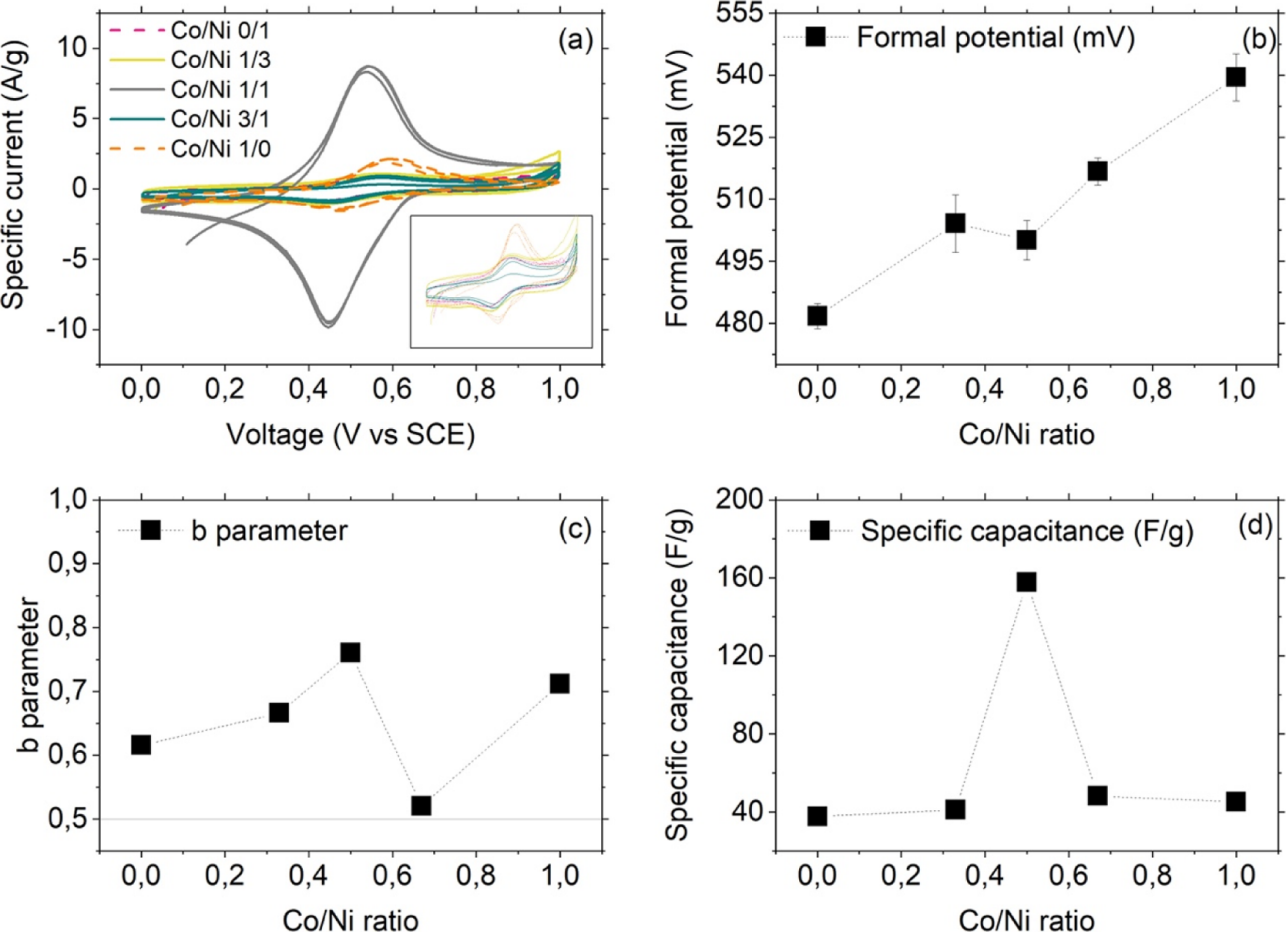
(a) CV curves (scan rate = 100 mV/s) of the PBAs under investigation (the inset shows a zoom-in of the curves pertaining to all samples but the analogue Co/Ni 1/1). (b) Formal potential, (c) b parameter, and (d) specific capacitance as a function of Co/Ni ratio.

**Table 6:** Formal Potential, Charge Storage Control *b* Parameter, and Specific Capacitance, as Retrieved from CV Measurements

Co/Ni ratio	formal potential *E* ^0^′ (mV) in K_2_SO_4_	*b* parameter		specific capacitance (F/g)
		Na_2_SO_4_	K_2_SO_4_	K_2_SO_4_
0/1	481.7 ± 3.0	0.6143 ± 0.00049	0.6156 ± 0.01631	37.7
1/3	504.1 ± 7.0	0.6208 ± 0.0072	0.6662 ± 0.02139	41.2
1/1	500.1 ± 4.8	0.6213 ± 0.1621	0.7606 ± 0.00428	157.7
3/1	516.7 ± 3.3	0.5485 ± 0.0415	0.5260 ± 0.04204	48.2
1/0	539.5 ± 5.7	0.4097 ± 0.0380	0.7114 ± 0.01561	45.3

An increase of the formal potential was recorded with increasing Co content in the samples, with no significant difference between the specimens featuring a Co/Ni ratio of 1/3 and 1/1, testifying to the ease of the oxidation Co^II^ → Co^III^.

All samples displayed good faradaic and charge storage behavior, shown by the increase of the area covered by the CV curves and peak intensity with increasing scan rate (Figure S4 in the Supporting Information).

The separation between the anodic and cathodic peaks (reported in Figure S6 in the Supporting Information) was observed to be significantly smaller for the t-PBA with Co/Ni = 1/1, compared to the others, indicating a higher degree of reversibility.

Obvious differences in the current density associated with the applied voltage can be seen among the samples (Figure 7a): the rate of charge transfer is a function of material composition. However, no obvious trend could be identified. The ternary analogue with a Co/Ni ratio equal 1/1 overperformed the other materials by ten times in terms of charge transfer rate and demonstrated superior capacitance (Table 6). The other composites displayed similar transfer rate, with the binary Co analogue performing slightly better than those containing Ni.

Due to the interest PBAs retain as potential supercapacitors, we evaluated the charge storage control mechanism based on CV outcomes, by using the following equation: *J* = *k*ν^*b*^, expressing the dependence of the current density *J* on the scan rate ν, where *k* is an experimental parameter and the exponent *b* can vary between 0.5 and 1 (Figure 7c,a). The closer *b* is to 1, the more capacitive the charge storage is, while the closer *b* is to 0.5, the more kinetically controlled the charge storage control is. *b* parameter was then obtained by extrapolation of the trend of *J* vs the scan rate.^26^

Changes in material composition are reflected in the charge storage control mechanism. Specifically, we recorded an increase in the capacitive charge storage going from Co/Ni ratio of 0/1 to 1/1. The lowest value was recorded for the sample with Co/Ni = 1/3, which, however, displayed also some instability upon voltage scans (Figure S4d in the Supporting Information).

Literature investigations on b-PBAs reported mixed charge storage control mechanisms, i.e., both capacitive and diffusive, which are herein confirmed also for the ternary analogues.^27^

The specific capacitance of the analogues was calculated as well as a function of the Co/Ni ratio (Table 6). Remarkably, we did not observe significant differences ascribable to the changes in material composition, with the exception of the ternary analogue featuring a Co/Ni ratio of 1/1, which showed neatly enhanced value compared to those recorded for the other compounds.

Capacitance retention and material stability upon voltage solicitation were evaluated by CV upon 2000 cycles (scan rate 100 mV/s). Figure 8a–e shows cycles 1 to 100 and 900 to 1000 (we omitted the other cycles for clarity reasons): great stability was recorded for the binary Ni analogue, with capacitance retention as high as more than 80%. This finding is attributed to the almost inertial electrochemical behavior of Ni^2+^, which is considered only a spectator during the voltammetric measurements, not participating in charge exchanges. Similar considerations can be done for the ternary analogue Co/Ni 1/3, whose current–voltage profile was almost unchanged after 1000 cycles. The slightly higher capacitance recorded for this sample, as compared with the binary Ni PBA, is ascribed to the action of the Co ions added to the crystal structure, which should promote better charge storage (and more capacitive, as proven by the higher value of the *b* parameter).

**Figure 8:**
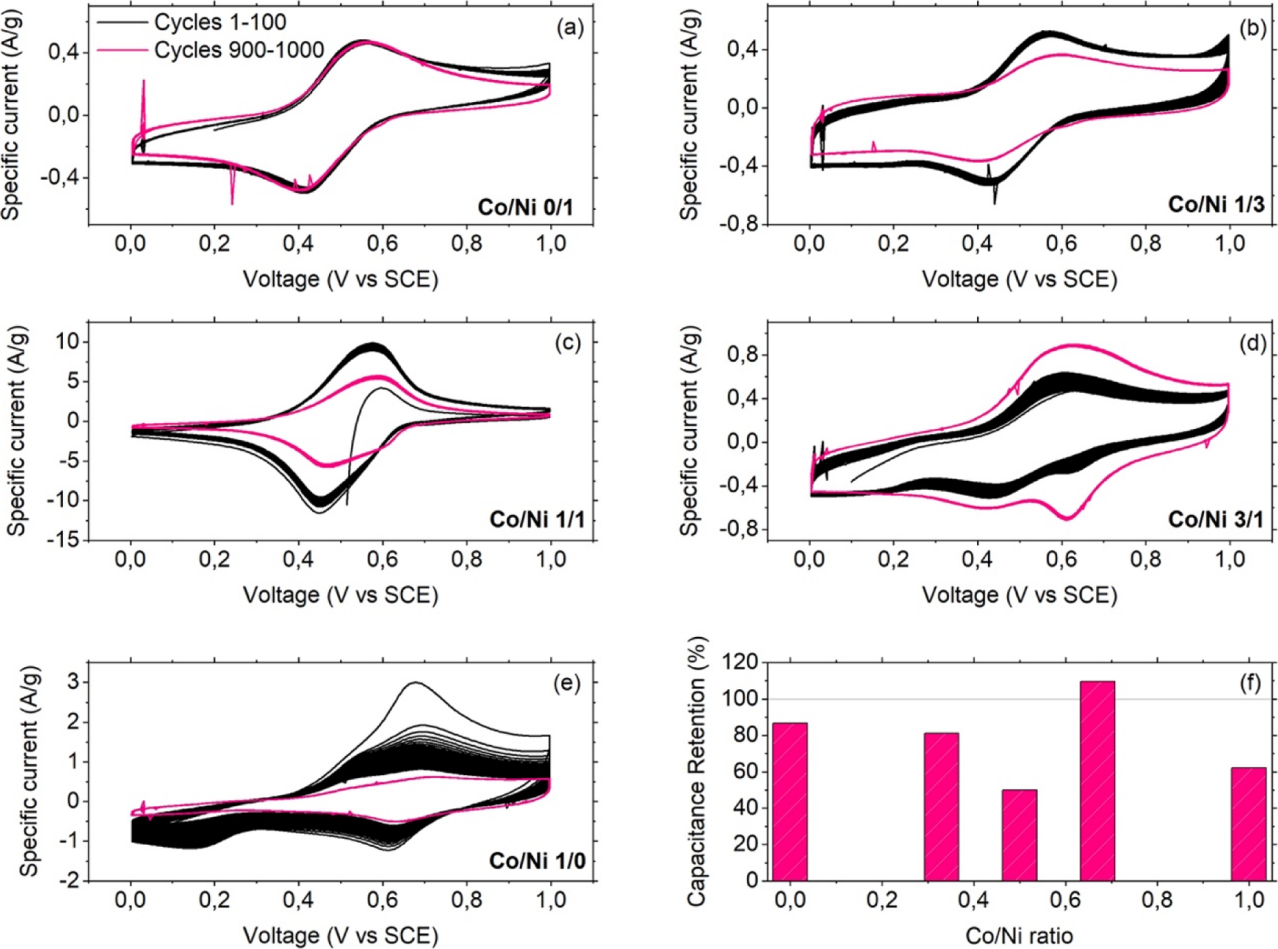
(a–e) Voltammetric cycles 1–100 (black line) and 900–1000 (pink line) for the PBAs under analysis. (f) Capacitance retention percentage after 1000 cycles as retrieved from CV measurements versus Co/Ni ratio.

Further increase of the Co content significantly changed the long-term behavior of the specimens. The t-PBA with an equal amount of Ni and Co (Figure 8c) conserved rather well the original current–voltage figure, with a decrease in the area circumscribed by the curves recorded at different cycles and the appearance of a shoulder centered at about 615 mV prior to the reduction peak, which became a real peak upon the further increase of Co/Ni ratio to 3/1 (Figure 8c), indicating the occurrence of another electrochemical process. The same effect was observed in the binary Co analogue, for which this contribution became the only reduction process over time. It is acknowledged that PBAs can insert/desert alkali cations,^28^ with a process efficiency depending, among other parameters, on the nature of the metals. We then checked the behavior of the materials using a 0.5 M aqueous solution of Na_2_SO_4_ (results pertaining to the binary Co analogues are shown in Figure S5 in the Supporting Information; *b* parameter is reported in Table 6 for comparison purposes), where no sign was found of the peak centered at 615 mV.

Based on this and on previous literature findings,^29^ we identified the following redox process:

(i) The main current peaks are ascribed to the insertion/desertion of the alkali metals in/from the crystal structure of all the analogues, with the concomitant redox process of the Fe^III^/Fe^II^ couple: they are found in both electrolytes, and the presence of Co does not play a major role;
(ii) Minor redox contributions are observed around 0.2 V vs SCE for the binary Co analogue and the ternary sample with Co/Ni 3/1: these are due to the interplay Fe^II^ ↔ Fe^III^ and Co^II^ ↔ Co^III^;
(iii) The cathodic contribution at higher potential (>600 mV vs SCE) is due to the formation, over time, of another form of Co hexacyanoferrate, with both transition metal ions in oxidation state + II, similar to the formation of Everitt’s salt from PB reduction, with a different ratio K/Co, which is electroactive at a different potential.^30,31^ This is confirmed by the observation that the only analogue not showing any contribution is the binary Ni PBA (due to the electrochemical “inertia” of Ni^II^) and that the higher the Co amount, the more pronounced the reduction contribution.

A more detailed analysis was reported in a dedicated investigation by Lezna and co-workers,^29^ who also highlighted the great complexity of Co hexacyanoferrates.

The t-PBA with a Co/Ni ratio of 3/1 displayed the most interesting behavior in long CV cycling, increasing its capacitance retention; this is ascribed to a change in material structure, testified by the relevant increase in current contribution of the peak centered at 615 mV, with the concomitant decrease of the contribution at lower potential (well visible in Figure 8d).

#### 3.4.2 Galvanostatic Charge–Discharge

Figure 9a,b report the GCD curves recorded for all of the materials at 25 and 250 mA/g, respectively. Obvious differences can be appreciated at 25 mA/g (Figure 9a) as a function of the Co content in terms of discharge kinetics, which are found to be slower for higher Co amounts, indicating an increase in the specific capacity. Imposing a ten times higher current (Figure 9b) resulted in a drastic change in the behavior of the samples. The binary analogue CoHCF and the ternary analogue with Co/Ni as high as 1/1 still displayed a symmetric curve, with discharge kinetics about 1 and 3 min, respectively. Any difference was instead lost for the other analogues, including the binary NiHCF, which was charged and discharged in 5 s.

**Figure 9:**
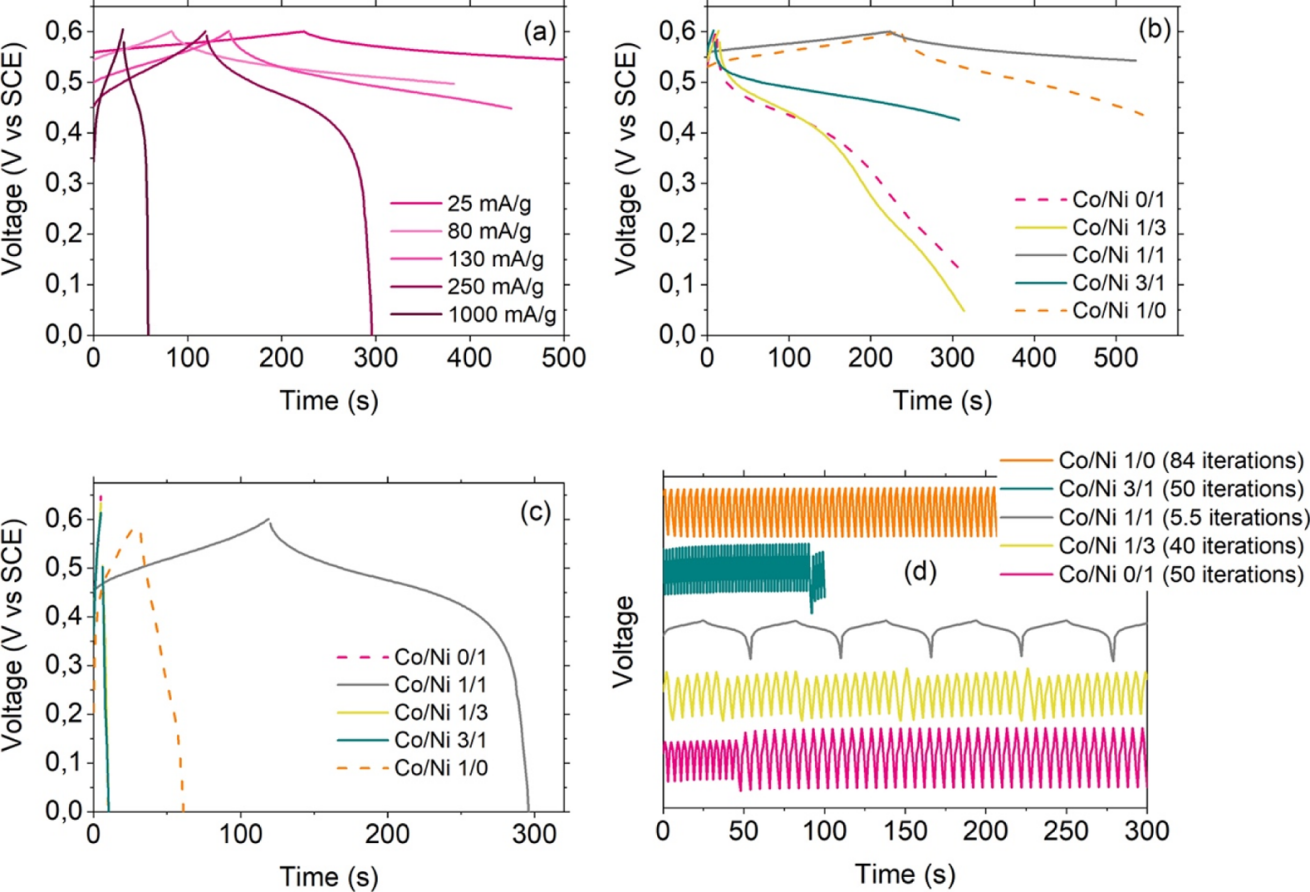
Galvanostatic charge–discharge curves in K_2_SO_4_: (a) and (b): comparison between the materials upon investigation at 25 mA/g and 250 mA/g, respectively; (c) sample with Co/Ni 1/1 at different currents. (d) GCD curves at 1 A/g recorded for 300 s; the legend reports the number of iterations for each sample. Sample Co/Ni 3/1 performed 50 iteration in 100 s.

The analysis of GCD curves pertaining to the ternary analogue Co/Ni 1/1 (Figure 9c) revealed a nice symmetry in time for all the applied currents, with an obvious reduction in the time needed to charge/discharge with the increase in current solicitation (the GCD curves at different currents pertaining to the other samples in the batch are shown in Figure S6 in the Supporting Information).

Testing the GCD rate and stability of the samples (we show the results for an applied current of 1 A/g and a charge–discharge time of 300 s in Figure 9d) proved once again the superior performance of the ternary analogue with equal amounts of Co and Ni: the charge–discharge curves retained their symmetry over time, with the slowest discharge rate.

It is also relevant to mention the Ohmic losses recorded for single GCD measurements (displayed in Figure S7 in the Supporting Information), which were observed to decrease as a function of the Co content, with a particularly good performance of the sample featuring the same amount of Ni and Co. Remarkably, for this latter, increasing ten times the current did not result in a drastic increase of the Ohmic loss. Ohmic loss during the GCD can be fitted as a function of the absolute current applied for all samples with a line whose slope correlates with the “sensitivity” of each material toward an increase of this stimulus (data and fits are shown in Figure S8 in the Supporting Information).

## 4 Discussion

The present study investigated the effect of varying the nominal composition of a series of binary and ternary hexacyanoferrates based on Ni and Co on their physical features and electrochemical performance. We showed that the well-established coprecipitation method, without the addition of any template or surfactant, is suited to modulate *on demand* the relative amount of Ni and Co, resulting in a batch well behaved from a structural, compositional, and chemical standpoint. The choice of water as the synthesis solvent returned specimens embedding water molecules of different kinds in the crystal structure, with the ternary analogues featuring smaller amounts of coordinated water compared with the binary analogues (Figure S1 and Table S1 in the Supporting Information). The crystal structure determined for the b-PBAs was not changed upon the fabrication of ternary analogues, and the unit cell size was barely affected, but the increase in Co content showed a very favorable effect in terms of the extension of the crystallite sizes (Table 1 and Figure 3). This also systematically affected the strength of the bond cyanide-iron and the relative amount of Fe^II^/Fe^III^ (Figure 3). This is a relevant parameter in the electrochemical behavior of the PBA family, i.e., eventually, on their potential as functional materials in applications where charge exchange plays a relevant role.

Ni was chosen as the third transition metal ion due to its electrochemical inertia: our initial assumption was that it should be in charge of guaranteeing stability to the crystal lattice under electrochemical stress, while Co and iron would play the role of active electrochemical agents. This hypothesis actually found confirmation in the long CV measurements (Figure 8): the samples containing the higher Ni amount did not show the emergence of any new species upon bias solicitation, indicating a more resistant lattice.

However, this was uncorrelated with the electrochemical performance: the current generated upon bias scanning did not show a trend with the sample composition (Figure S3 in the Supporting Information), where the sample with Co/Ni 1/1 overperformed the others roughly 8 times.

Moreover, this sample displayed better reversibility, higher capacitive behavior, and higher resilience toward galvanostatic charge/discharge, but at the same time lower capacitance retention upon potential cycling.

It is interesting to note that, while writing this study, we came to know that very similar results were reported by Yang and co-workers,^32^ who investigated a series of Co–Ni–Fe trimetallic PBA as electrochromic materials and found the sample with nominal Co/Ni ratio 1/1 performing the best, without, however, being able to fully justify the reasons behind the observed behavior.

Attempts to correlate the amount of water molecules in the PBA structure were previously made in literature, but in the present study we did not ascertain the same.^33^

## 5 Conclusions

In this study, ternary PBAs Ni–Co–Fe with different ratios of Co/Ni were prepared and characterized.

The materials were tested without any postsynthetic treatment (apart for a mild drying process) to investigate their intrinsic electrochemical behavior and their potential as supercapacitors.

Significant differences were recorded among the analogues, but no systematic trend as a function of the Co/Ni ratio was observed.

The outcomes of the present investigation showed that modulating the relative amount Co/Ni indeed plays a role in driving their electrochemical behavior, with unexpected results in terms of performance. The best balance between these two transition metal ions was found to be 1/1, which guaranteed the best electrochemical reversibility accompanied by the highest specific capacitance, the highest resilience toward high current solicitations, but at the same time the worst capacitance retention under potential stimulus.

This work is aimed at questioning (from a constructive perspective) the current trend in the literature about the use of PBAs as energy storage materials. We confirmed the complexity of these materials through their structural and physicochemical characterization, often highlighted in scientific papers previously appearing in the literature, presenting fundamental research, and, although their potential as electrochemical energy storage devices is undeniable, we believe that more care should be used when testing them as capacitive elements: without a deep investigation of their composition, oxidation states of the metals, water content, and coherent domain extension, we might not know what we are really measuring. Last but not least, investigations on the thermodynamics and kinetics of nucleation are definitely needed: the process is indeed still unclear, as proven by significantly different results in the literature while using the same synthetic approach.

## Supplementary Material



## Notes

Thermal analyses; SEM–EDX analysis; FTIR spectra and correlation tables of potassium hexacyanoferrate and PB; CV recorded in K_2_SO_4_; CV of specimen with Co/Ni ratio 1/0 recorded in Na_2_SO_4_; anodic–cathodic peak separation; and GCD curves (PDF)
