# Supplementary material for: Synthesis, Characterization, and Electrochemical Behavior of Ternary Ni–Co–Fe Prussian Blue Analogues
Source: ACS Omega. 2025 Aug 19;10(37):42980–92. doi: 10.1021/acsomega.5c05763 (PMC12461328; doi:10.1021/acsomega.5c05763)
Supplement: Supplementary file 1 [file ao5c05763_si_001.pdf]

Synthesis, characterization and electrochemical behavior of ternary Ni-Co-Fe Prussian blue analoguesTuning ternary Ni-Co-Fe Prussian blue analogues composition to understand their electrochemical behavior: a case study

Isabella Concina[a*], Janna Attari[a], Farid Akhtar[a], Alessio Mezzi[b], Shujie You[a]

[a] Luleå University of Technology, Department of Engineering Sciences and Mathematics, 97187 Luleå, Sweden

[b] CNR-ISMN, Strada provinciale 35d, n.9 00010 Montelibretti, Italy

* Corresponding author: isabella.concina@ltu.se

Supporting Information

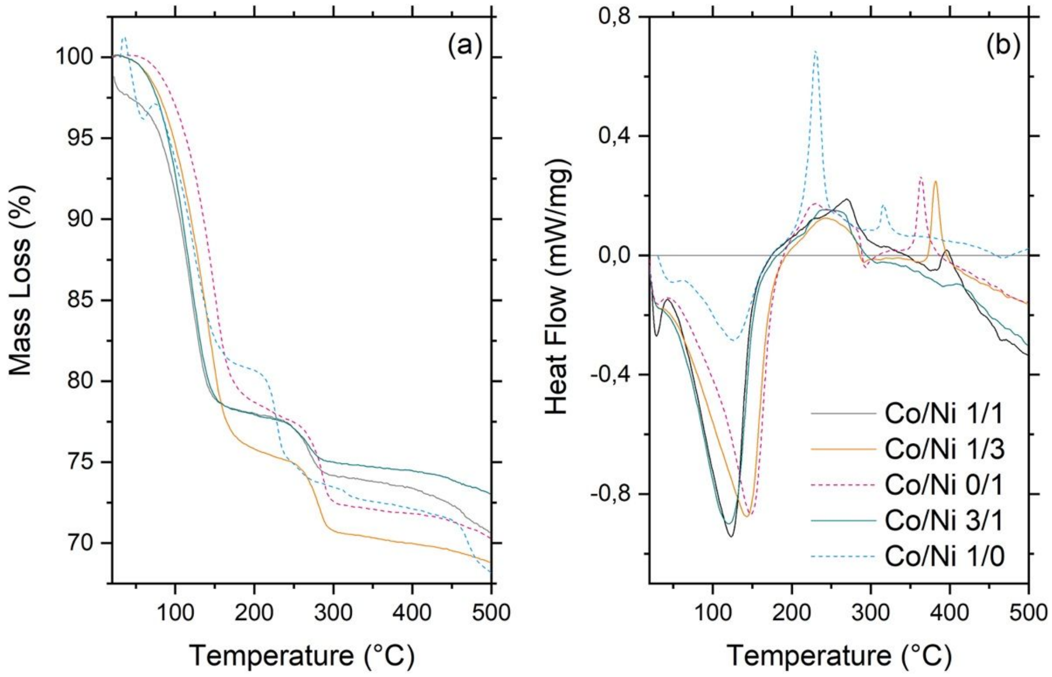

Figure S 1. (a) Thermal gravimetry and (b) differential scanning calorimetry (exo up) of the PBAs.

Co/Ni ratio	Sample label	Zeolitic water (mass %)	Coordinated water (mass %)	Total water content (mass %)
0/1	NiHCF	20.64	2.22	22.86
1/3	T2	23.65	1.35	25.00
1/1	T1	18.96	1.45	20.41
3/1	T4	21.48	1.02	22.50
1/0	CoHCF	15.54	3.68	19.22

Table S 1. Content of water in the Prussian blue analogues under investigation, as retrieved from thermal analyses.

Sample lable: T1	Co/Ni/Fe ratio:	1.5 / 1.4 / 2.0
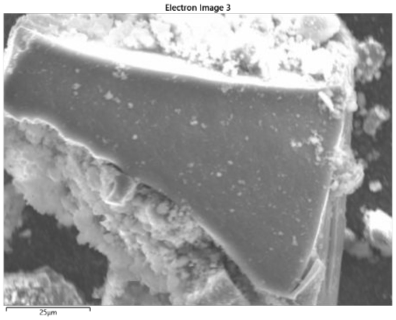	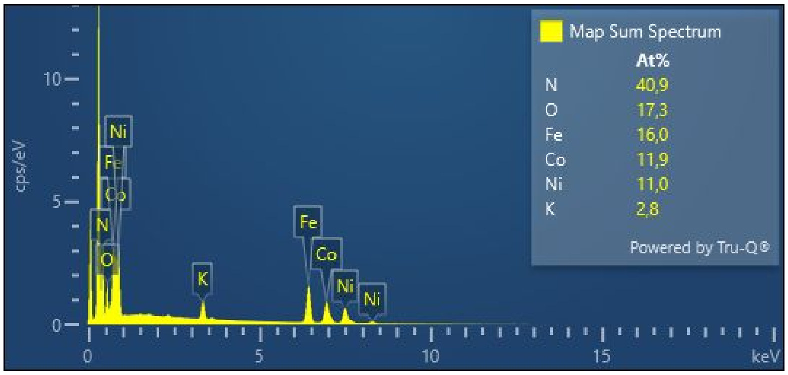	
Co Kα1	Ni Kα1	Fe Kα1
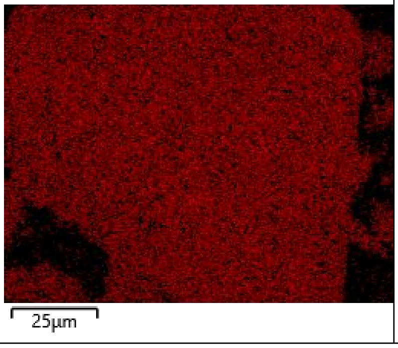	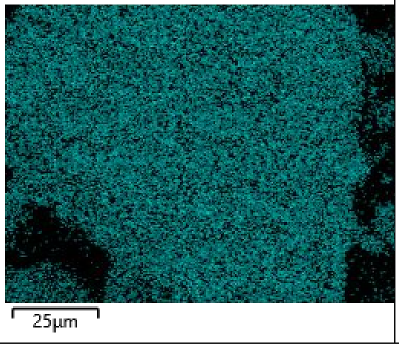	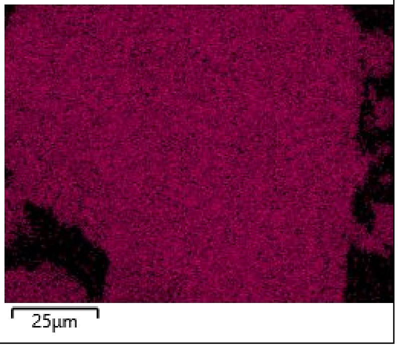

Sample lable: T2	Co/Ni/Fe ratio:	0.9 / 2.1 / 2.0
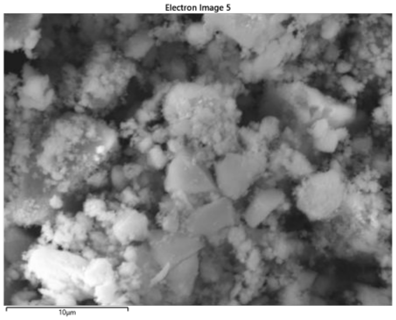	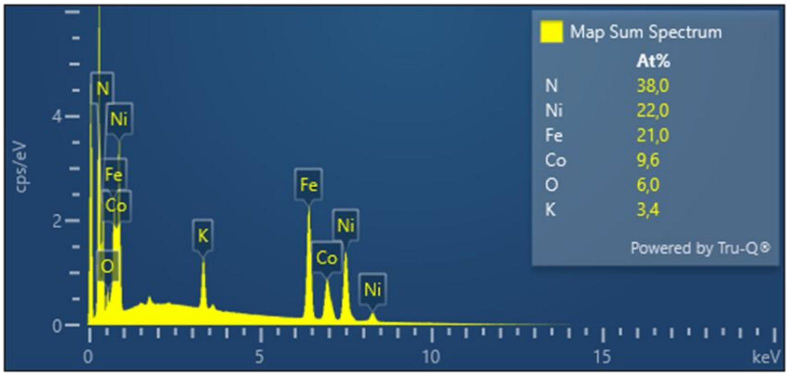	

<table>
<tr><td>Co Kα1</td><td>Ni Kα1</td><td>Fe Kα1</td></tr>
<tr><td>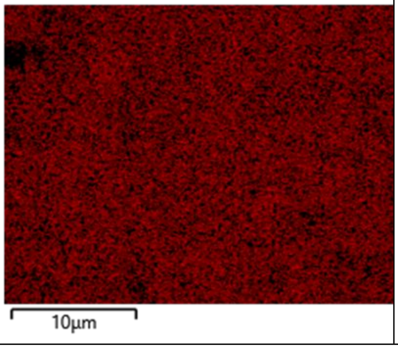
</td><td>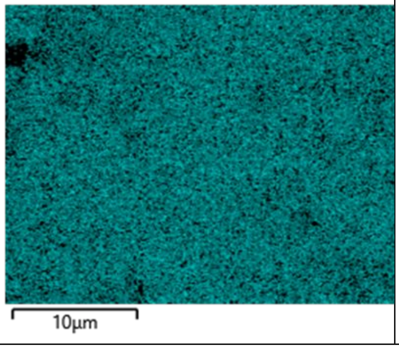
</td><td>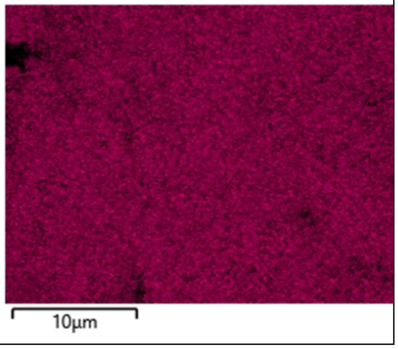
</td></tr>
</table>

<table>
<tr><td>Sample lable: T3</td><td>Co/Ni/Fe ratio:</td><td>0 / 3.1 / 2.0</td></tr>
<tr><td>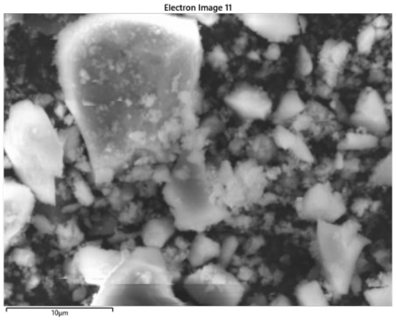
</td><td colspan="2">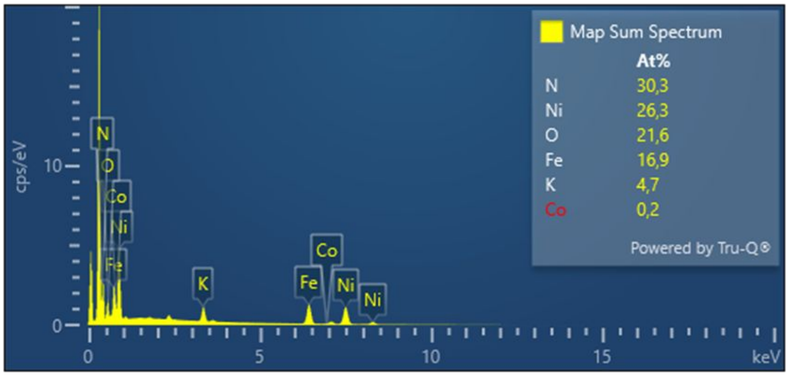
</td></tr>
<tr><td>K Kα1</td><td>Ni Kα1</td><td>Fe Kα1</td></tr>
<tr><td>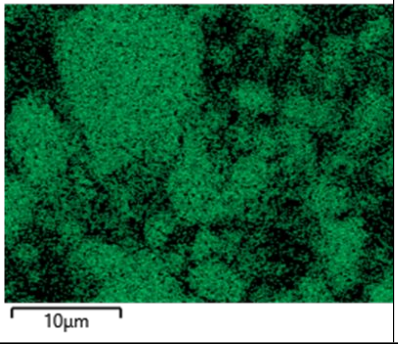
</td><td>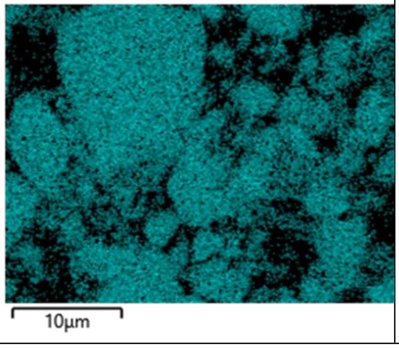
</td><td>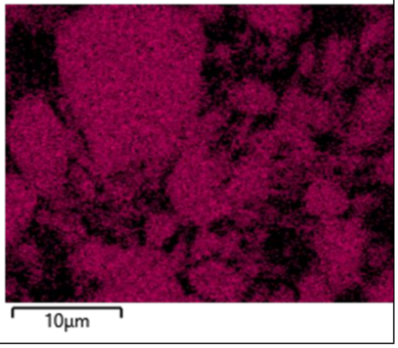
</td></tr>
</table>

<table>
<tr><td>Sample lable: T4</td><td>Co/Ni/Fe ratio:</td><td>2.0 / 0.8 / 2.0</td></tr>
<tr><td>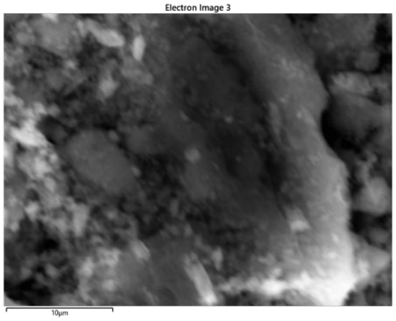
</td><td colspan="2">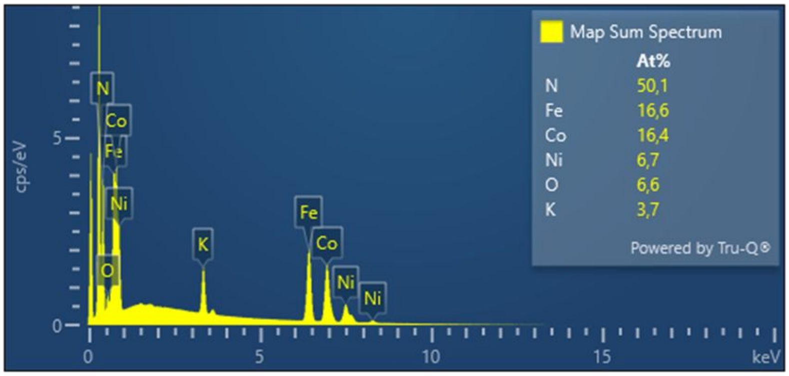
</td></tr>
<tr><td>Co Kα1
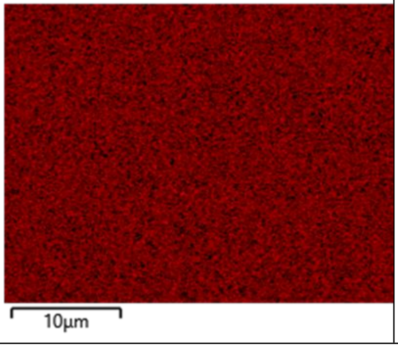
</td><td>Ni Kα1
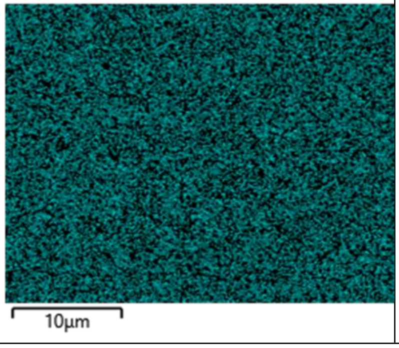
</td><td>Fe Kα1
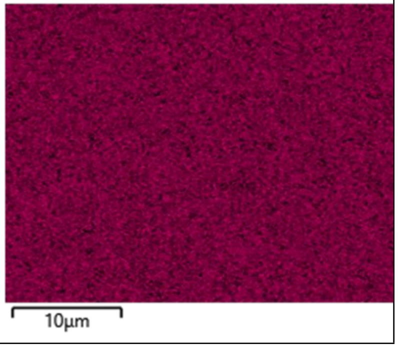
</td></tr>
</table>

<table>
<tr><td>Sample lable: CoHCF</td><td>Co/Ni/Fe ratio:</td><td>0 / 3.1 / 2.0</td></tr>
<tr><td>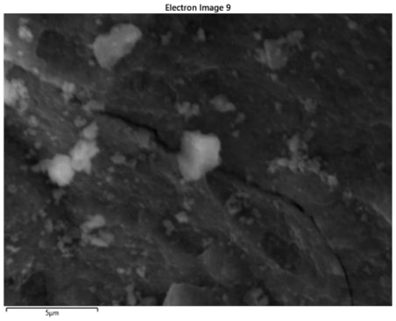
</td><td colspan="2">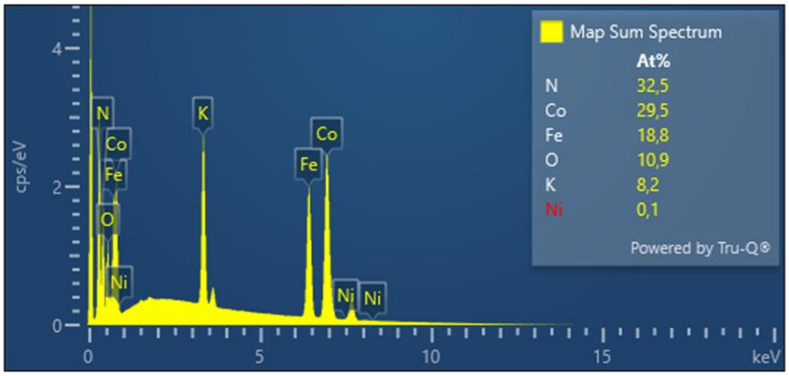
</td></tr>
<tr><td>Co Kα1
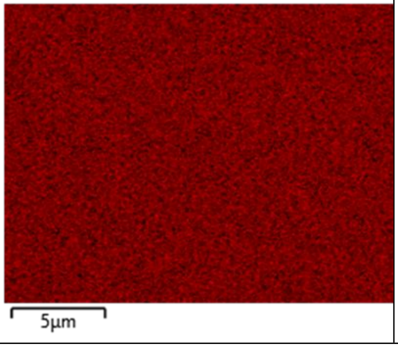
</td><td>K Kα1
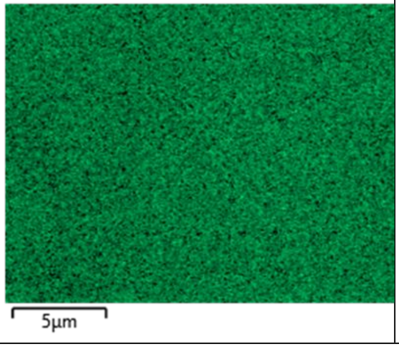
</td><td>Fe Kα1
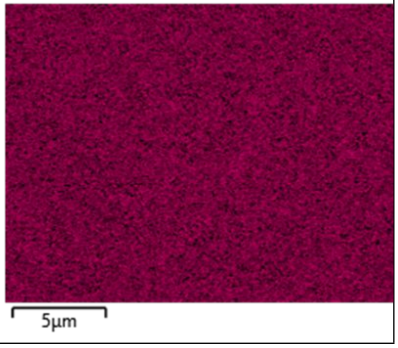
</td></tr>
</table>

Table S 2. EDX analysis.

**Co/Ni ratio →**	**0/1**	**1/3**	**1/1**	**3/1**	**1/0**	**Vibration**
**Sample Label →**	NiHCF	T2	T1	T4	CoHCF	
**Wavenumber (cm^{-1}) →**	3645	3645	3651	3647.2	3648	ν H_2O coord
	3375	3375	3324	3400	3354	ν H_2O H-bond
	2158.2 sh	2168.8<br>2164.9	2168.8	2166.9	2163	ν CN Fe^{III}
	2103.2<br>2096.5	2118.7<br>2096.5	2118.7<br>2102.3	2116.8<br>2101.3	2116.8<br>2095.5	ν CN Fe^{II}
	1650.0 sh	1650.0 sh	1671.2	1666.4 sh	1666.4 sh	δ H_2O H-bond
	1611.4	1611.4	1610.5	1611.4	1611.4	δ H_2O coord
	1410	1408.9	1415.7		1431.1	
	1343.3	1346.2	1372.3	1366.5	1343.3	
	1242.1	1240.2	1239.2<br>vw	1240.2	1232.5 w,<br>br	
	1043.4 vw				1047.3	

Table S 3. FTIR correlation table. ν = stretching, δ = bending, sh = shoulder, w = weak, vw = very weak, br = broad.

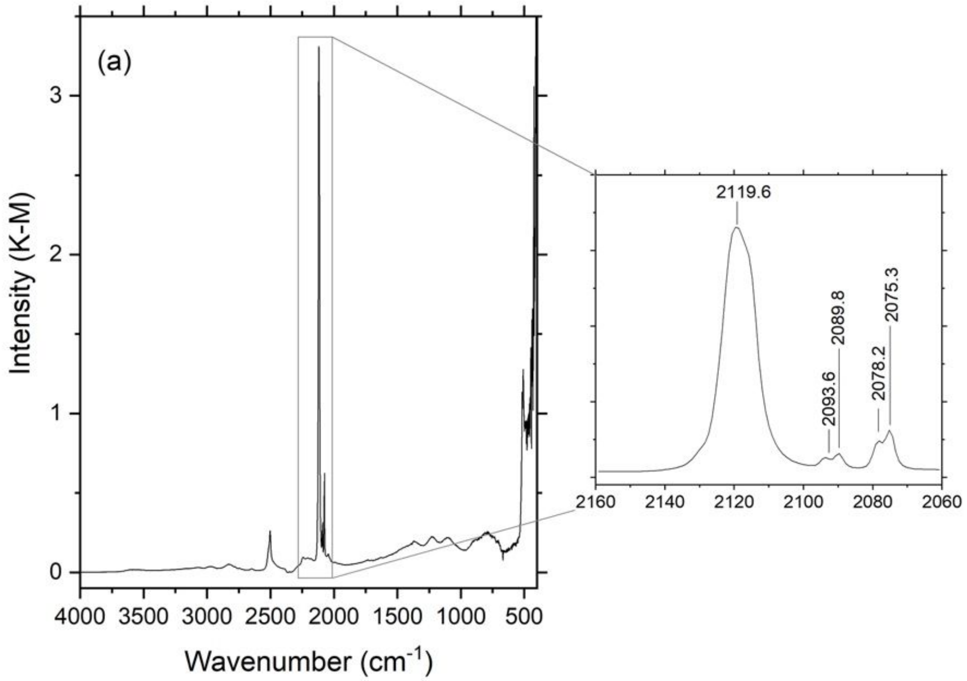

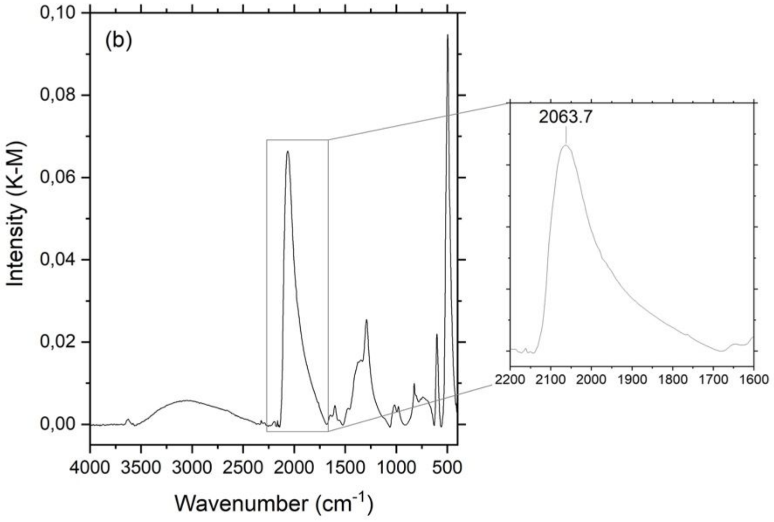

Figure S 2. FTIR spectra of the iron precursor $K_3[Fe(CN)_6]$ (a) and of Prussian blue (b).

**Sample →**	**$K_3[Fe(CN)_6]$§**	**Vibration**	**Prussian blue‡**	**Vibration**
**Wavenumber (cm^{-1}) →**	2503,5 w	Combination stretch CN<br>$\nu_3 + \nu_8$<br>$\nu_1 + \nu_2$	3624.0	$\nu\ (H_2O)_{coord}$
	2119.6 vs	ν_6 CN	2162.0 vw	ν CN(Fe^{III})
	2093.6 w<br>2089.8 w	ν_6 CN^{15}	2061.7	ν CN(Fe^{II})
	2078.2<br>2075.3	ν_6 $C^{13}N$		
	1372.3 w<br>1231.5 w<br>1109.0 v	Not assigned	1645.2 w	$\delta\ (H_2O)_{HB}$
	793.7	Combination<br>$N_2 + \nu_8$	1600.8 w	$\delta\ (H_2O)_{coord}$
	516.9<br>508.2	ν_7 Fe-C	599.8 s<br>513.0 sh	δ Fe^{II}-C<br>ν Fe^{II}-C

§ Vibrations assigned according to the spectral analysis reported in J.T.R. Dunsmuir, A.P. Lane, The infrared spectra (40-3000 cm-1) of potassium hexacyanoferrate and potassium hexacyanocobaltate, J. Chem. Soc. A Inorganic, Phys. Theor. Chem. (1971) 776–780. https://doi.org/10.1039/J19710000776.

‡ Vibrations assigned according to the spectral analysis reported in S.G. Khasevani, D. Nikjoo, D.O. Ojwang, L. Nodari, S. Sarmad, J.-P. Mikkola, F. Rigoni, I. Concina, The beauty of being complex: Prussian blue analogues as selective catalysts and photocatalysts in the degradation of ciprofloxacin, J. Catal. 410 (2022) 307–319. https://doi.org/10.1016/j.jcat.2022.04.029.

Table S 4. FTIR correlation table for the iron precursor $K_3[Fe(CN)_6]$ and Prussian blue. W: weak; vw: very weak; s: strong; sh: shoulder; coord: coordinated; HB: hydrogen-bonded.

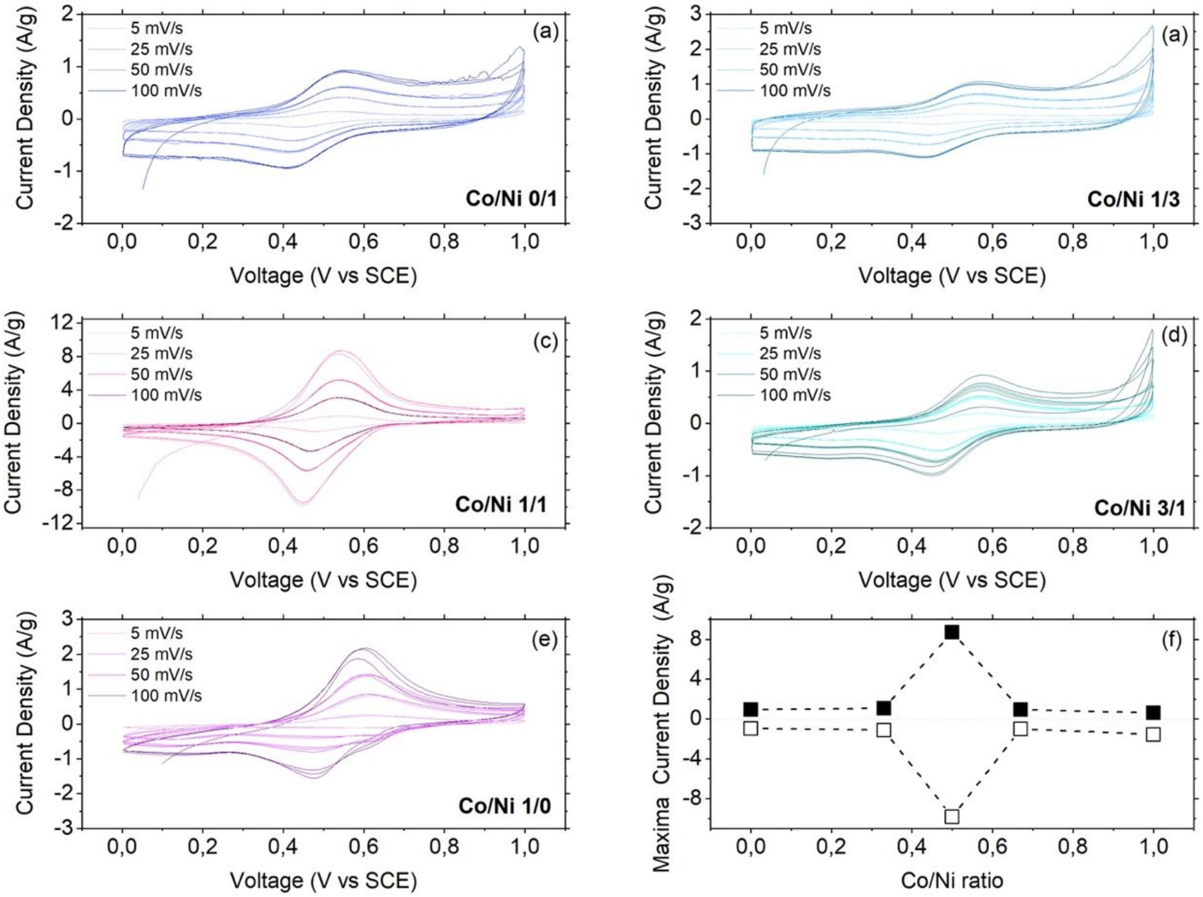

Figure S 3. Cyclic voltammograms (a-e) recorded in K_2SO_4 for all the analogues under investigation. (f) Current density anodic and cathodic peaks values as a function of Co/Ni ratio, as retrtieved ffrom the CV measurements (scan rate: 100 mV/s).

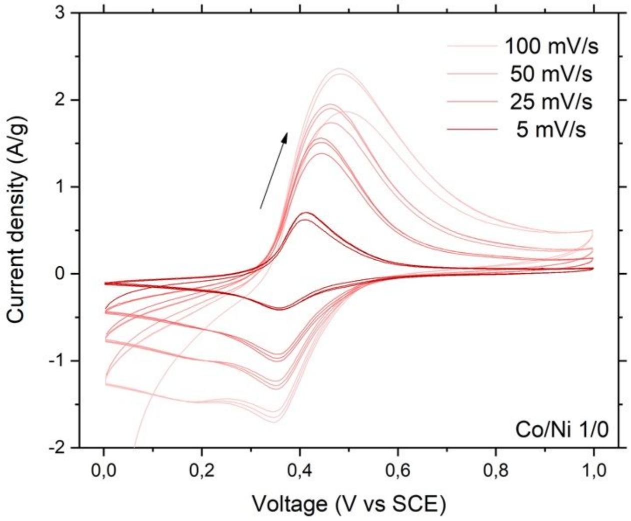

Figure S 4. Cyclic voltammetric measurements of binary cobalt hexacyanoiferrate in Na_2SO_4 electrolyte.

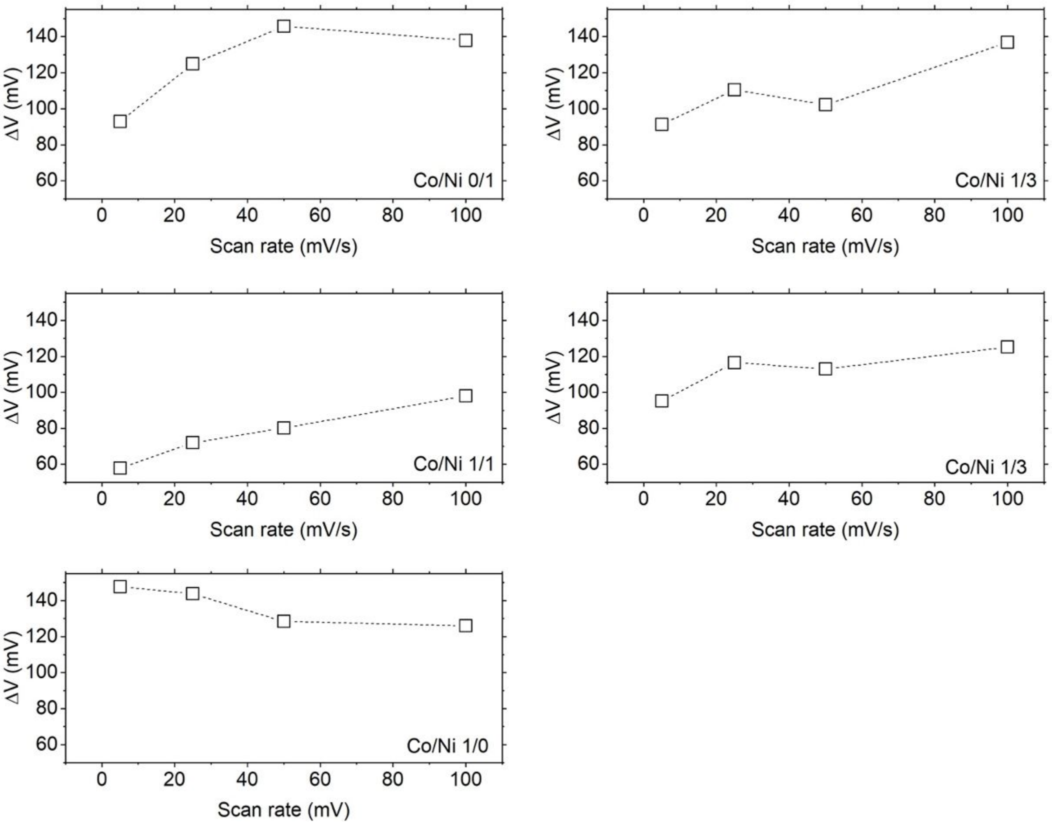

Figure S 5. Anodic-cathodic peak separation in K_2SO_4.

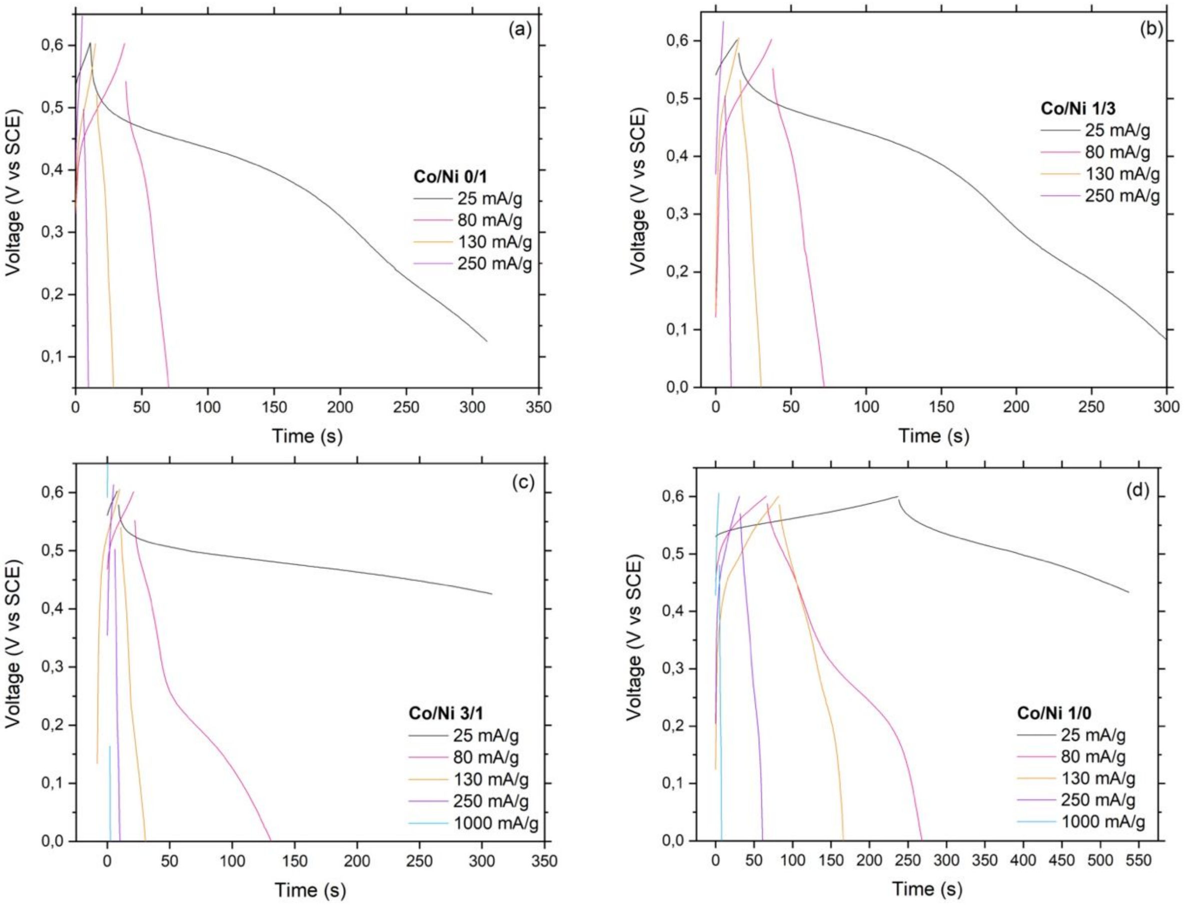

Figure S 6. Galvanostatic charge discharge measurements for the samples under investigation. (a). Co/Ni ratio 0/1; (b) Co/Ni ratio: 1/3; (c) Co/Ni ratio 1/1; (d): Co/Ni ratio 1/0.

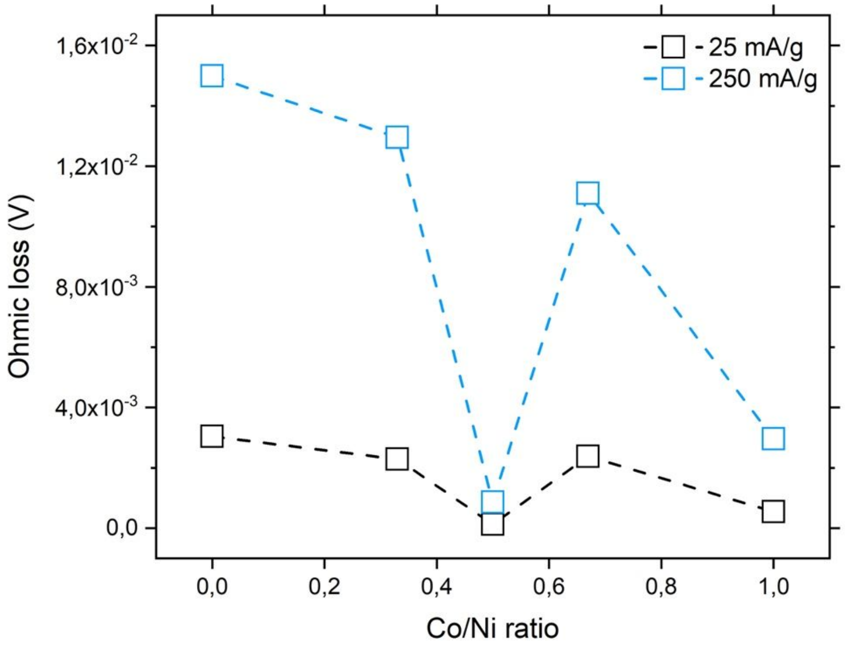

Figure S 7. Ohmic loss at 25 and 250 mA/g as a function of Co/Ni ratio.

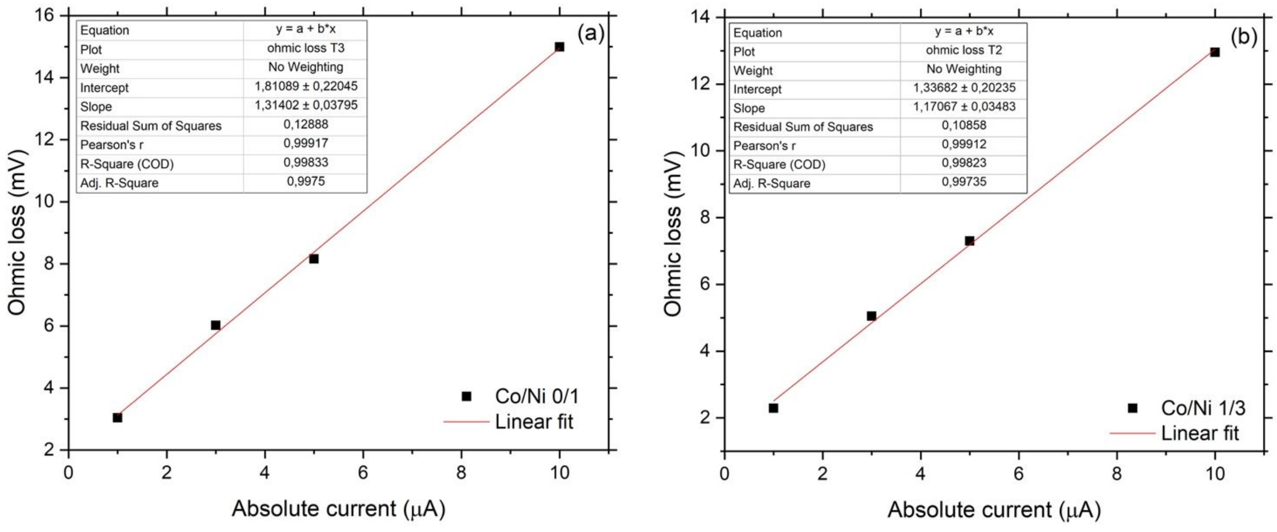

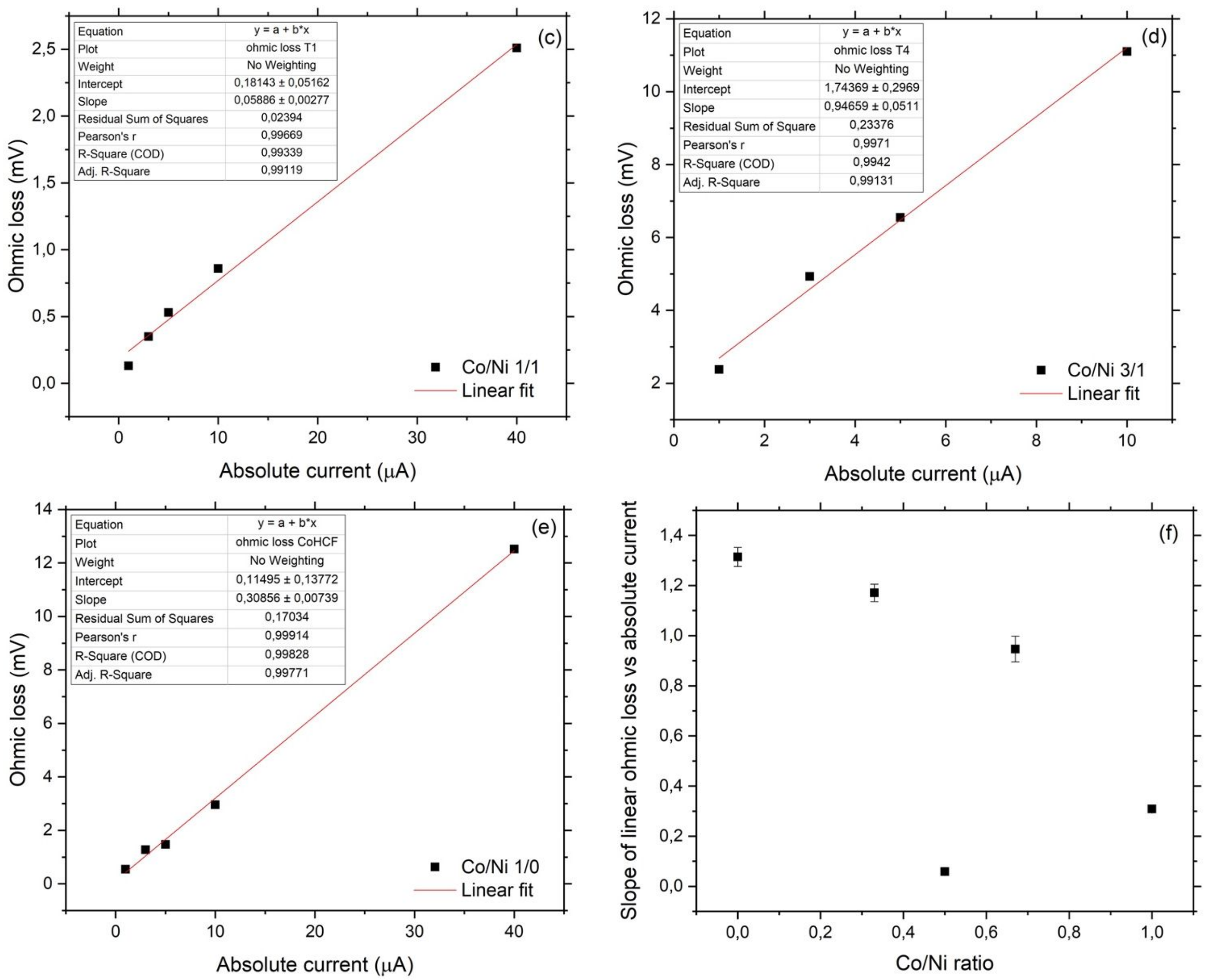

Figure S 8. (a)-(e): Ohmic loss as a function of the applied absolute current. (f) Dependence of ohmic loss on the materials composition, as retrieved from the linear fitting.
